# Lifespan Extension by Preserving Proliferative Homeostasis in *Drosophila*

**DOI:** 10.1371/journal.pgen.1001159

**Published:** 2010-10-14

**Authors:** Benoît Biteau, Jason Karpac, Stephen Supoyo, Matthew DeGennaro, Ruth Lehmann, Heinrich Jasper

**Affiliations:** 1 Department of Biology, University of Rochester, Rochester, New York, United States of America; 2 Howard Hughes Medical Institute and Kimmel Center for Biology and Medicine of the Skirball Institute, Department of Cell Biology, New York University School of Medicine, New York, New York, United States of America; Stanford University Medical Center, United States of America

## Abstract

Regenerative processes are critical to maintain tissue homeostasis in high-turnover tissues. At the same time, proliferation of stem and progenitor cells has to be carefully controlled to prevent hyper-proliferative diseases. Mechanisms that ensure this balance, thus promoting proliferative homeostasis, are expected to be critical for longevity in metazoans. The intestinal epithelium of *Drosophila* provides an accessible model in which to test this prediction. In aging flies, the intestinal epithelium degenerates due to over-proliferation of intestinal stem cells (ISCs) and mis-differentiation of ISC daughter cells, resulting in intestinal dysplasia. Here we show that conditions that impair tissue renewal lead to lifespan shortening, whereas genetic manipulations that improve proliferative homeostasis extend lifespan. These include reduced Insulin/IGF or Jun-N-terminal Kinase (JNK) signaling activities, as well as over-expression of stress-protective genes in somatic stem cell lineages. Interestingly, proliferative activity in aging intestinal epithelia correlates with longevity over a range of genotypes, with maximal lifespan when intestinal proliferation is reduced but not completely inhibited. Our results highlight the importance of the balance between regenerative processes and strategies to prevent hyperproliferative disorders and demonstrate that promoting proliferative homeostasis in aging metazoans is a viable strategy to extend lifespan.

## Author Summary

Somatic stem cells are critical for regeneration of many tissues, thus ensuring long-term maintenance of tissue function. Proliferation of stem and progenitor cells has to be limited, however, to prevent hyperproliferative diseases and cancer in aging animals. This conflict between the need for stem cell proliferative potential and cancer prevention compromises regeneration in many high-turnover tissues of aging animals, including humans. It remains to be established whether and how proliferative homeostasis can be optimized to positively influence lifespan. Our work addresses this question using fruitflies as a model, taking advantage of the recent discovery of regenerative processes in adult flies. In old flies, intestinal stem cells (ISCs) hyperproliferate, causing an accumulation of mis-differentiated daughter cells (a phenotype termed intestinal dysplasia). We show that the balance between regeneration and dysplasia in this tissue significantly influences lifespan. When ISC proliferation rates are reduced, but not completely inhibited, dysplasia is limited and lifespan is increased. This can be achieved by moderately reducing insulin and stress signaling activities, as well as by expressing protective proteins in somatic stem cell lineages. Our results show that optimizing proliferative homeostasis (i.e. limiting dysplasia, but allowing sufficient regeneration) in high-turnover tissues is an efficient strategy to extend lifespan.

## Introduction

Lifespan of many organisms can be increased by optimizing both genetic and environmental conditions, including reducing calorie intake [1]–[3], increasing oxidative stress protection [4], [5] and reducing Insulin/IGF1 signaling (IIS) [6]–[8]. These different interventions are likely to be acting through related mechanisms, notably by increasing stress-protective gene expression in differentiated somatic cells, prolonging their functional lifespan and delaying tissue degeneration [7], [9]–[12]. In addition to such stress-protective mechanisms, metazoans also maintain tissue homeostasis through regenerative processes that rely on the long-term maintenance of a functional population of somatic stem and progenitor cells. For these cells, a similar, and perhaps more significant, relationship between stress protection and lifespan is expected, as their long-term maintenance is critical to conserve regenerative capacity. This relationship is complicated, however, by the fact that such cells are mitotically active, and their deregulation thus has the potential to promote dysplasia and increase the incidence of cancer [13], [14]. Accordingly, mammalian stem cells generally exhibit a robust intrinsic ability to limit and repair intracellular damage [15]–[19], yet also employ strong anti-proliferative mechanisms that prevent cancer, but limit the regenerative capacity of stem cells in old age [18], [20]–[22]. The regenerative decline of many tissues is thus caused by oxidative stress and DNA damage in stem and progenitor cells, as well as by cell-autonomous up-regulation of cell cycle inhibitors like p16, and by changes in the systemic environment [13], [20], [23]–[27]. Accordingly, processes that maintain the regenerative capacity of stem and progenitor cell populations, but prevent hyper-proliferation and cancer (i.e. processes that promote proliferative homeostasis), are expected to significantly influence longevity of the organism [28].

Recent studies in mouse hematopoietic stem cells (HSCs) indicate that the IIS pathway and its downstream transcription factor Foxo constitute an important regulatory system that controls stem cell stress protection while also influencing proliferation [18], [29]–[32]. Foxo (Daf-16 in *C.elegans*) is repressed by IIS and is required for the lifespan extension observed when IIS activity is reduced either systemically, or specifically in adipose tissue [6]–[8]. Foxo induces the expression of genes involved in scavenging reactive oxygen species (ROS) and repairing damage to DNA and proteins, while also inducing cell cycle inhibitors [33]–[37]. Loss of Foxo in HSCs therefore results in increased proliferation of the HSC population, while boosting ROS levels and increasing apoptosis. As a consequence, the long term repopulating ability of HSCs is reduced [18], [29], [30], [32].

*Drosophila* is emerging as a genetically tractable model to assess the importance of regeneration in lifespan and aging [38]–[43]. Recently identified somatic stem cells in *Drosophila* include intestinal stem cells (ISCs) in the posterior midgut epithelium, as well as stem cells in malpighian tubules [44] and the hindgut [45], [46]. ISCs are critical for regeneration and maintenance of the midgut epithelium [47]–[50]. These cells are characterized by the expression of the marker genes *escargot* and *Delta*, and divide asymmetrically to give rise to a new ISC and an Enteroblast (EB) that differentiates into one of two cell types: Enterocytes (ECs) and Enteroendocrine cells (EEs). In contrast to the mammalian lineage, no transit amplifying cell population exists in *Drosophila*; ISC being the only dividing cell type in the midgut epithelium [47]–[49]. In young animals, ISCs divide rarely, as less than 5 mitoses can be observed at any given timepoint in the intestine [42], [47]–[49]. In response to stressful challenges, however, ISC proliferation is strongly increased, a regenerative response that allows restoring large parts of the intestinal epithelium in response to damaging agents, such as pathogens, genotoxins, or ROS inducing compounds [42], [43], [50]–[53]. Interestingly, this regenerative function of ISCs can have deleterious consequences for the organism, as excessive proliferation of ISCs in response to stress is accompanied by the accumulation of mis-differentiated cells in the intestine, which ultimately disrupts epithelial integrity with a dysplastic phenotype [43]. In the aging gut, such dysplasia is widely observed under normal culture conditions, suggesting that an age-related over-proliferation of ISCs (due to either elevated or chronic oxidative stress or to pervasive inflammation) contributes to the loss of intestinal function and to the increased mortality of aging flies [42], [43], [54]. This phenotype is caused by an age-related increase in the activity of the stress-responsive Jun-N-terminal Kinase (JNK) signaling pathway [43], [54].

The *Drosophila* intestine thus constitutes an accessible model system to study whether preserving proliferative homeostasis of aging tissues can influence overall lifespan of metazoans. We initiate such studies here by assessing the effects of intestinal dysplasia on lifespan. Our results reveal a significant correlation between the loss of proliferative homeostasis in the intestinal epithelium and fly lifespan. Importantly, we show that limiting proliferation rates by moderately reducing IIS or JNK activities in the somatic stem cells lineages is sufficient to extend lifespan. We further find that the beneficial effects of reducing IIS activity in this lineage can be recapitulated by selectively over-expressing stress-protective Foxo target genes. In such flies, intestinal dysplasia is delayed, accompanied by improved maintenance of metabolic health, and by increased lifespan. Our results demonstrate that promoting proliferative homeostasis in somatic tissues is sufficient to extend lifespan in metazoans.

## Results

### Intestinal regeneration influences lifespan

Recent studies suggest a significant influence of intestinal regeneration on fly viability. Flies in which intestinal dysplasia is accelerated are short lived [43], while animals with impaired ISC proliferation or daughter cell differentiation die faster when infected by enteropathogenic bacteria than wild-type flies [50], . These observations indicated that genetic conditions in which intestinal homeostasis is preserved might result in increased lifespan.

To start testing this hypothesis, we first tested the requirement of ISC-mediated tissue renewal for optimal lifespan. Ectopic activation of Notch signaling in ISCs was previously shown to irreversibly impair their function by promoting differentiation [43], [49]. In order to abolish ISC function, we thus transiently expressed an activated form of Notch (IntraCellular Domain; NICD) in ISCs and EBs using the esgGal4 driver. In young adult esgGal4 heterozygous flies, Gal4 activity is restricted to ISCs and EBs in the intestine, to malpighian tubule stem cells, as well as to the testis and salivary glands, and is not detected in other tissues (Figure S1). To prevent developmental effects of the expression of UAS-driven transgenes, we used a heat-inducible system in which esgGal4 is combined with a temperature-sensitive Gal80 (TARGET system, [55]; Figure S2D). Transient expression of NICD for 7 days in young flies, significantly shortens lifespan (Figure S2A), supporting the notion that maintaining somatic stem cell function is critical for optimal lifespan. Importantly, longevity is not significantly affected when these flies are kept at a permissive temperature throughout life, confirming that lifespan shortening is caused by transient adult expression of NICD, and not by ectopic expression of the protein during development (Figure S2B; further confirming the selective inducibility of the employed TARGET system, UAS-linked transgene expression is detectable in esgG4, tubGal80^ts^ flies only at the restrictive temperature, 29°C, Figure S2D, S2E).

**Figure 1:**
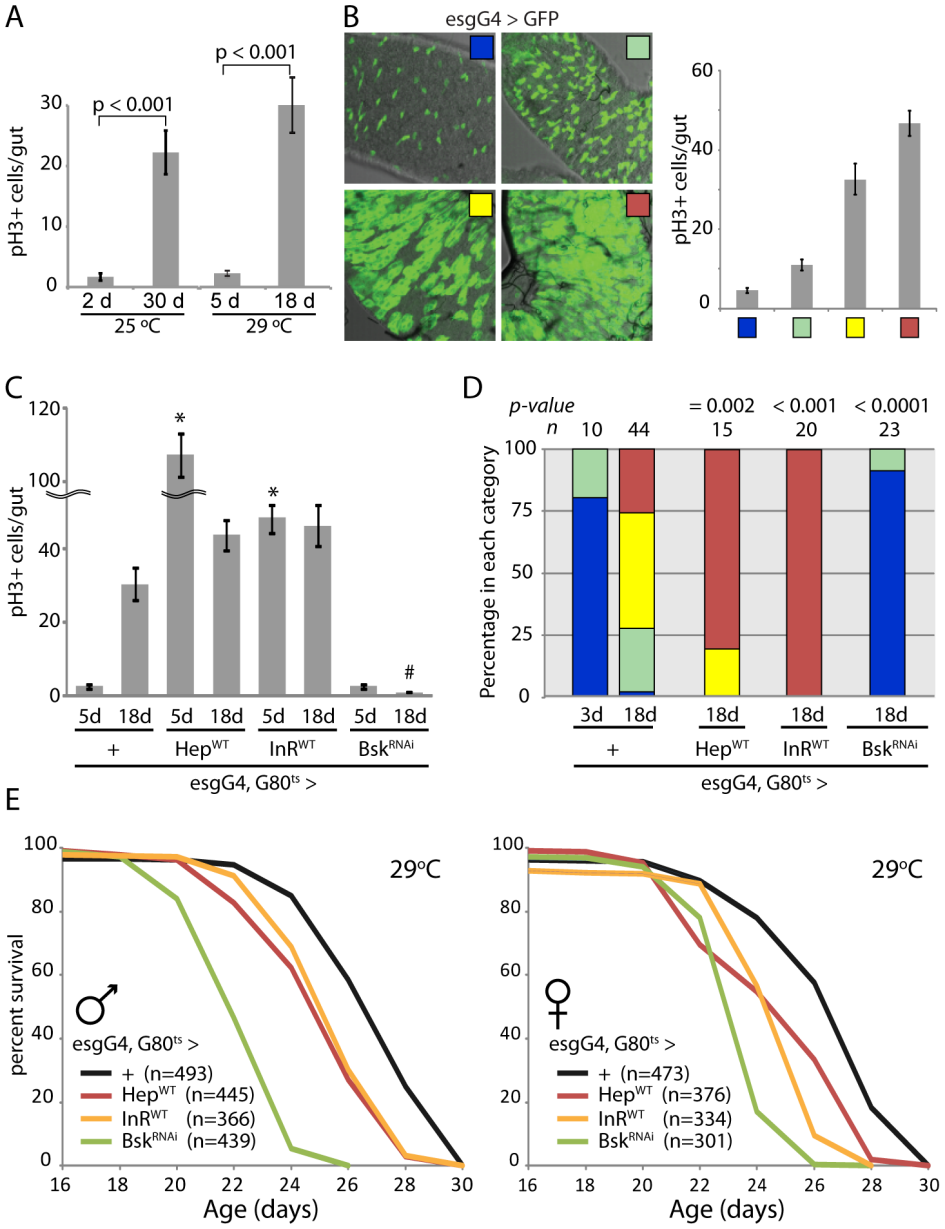
Intestinal homeostasis and tissue regeneration is critical for normal lifespan. A. Age-related increase in the frequency of pH3^+^ cells in the aging intestine of wild-type flies (Average and SEM is shown). ISC over-proliferation is accelerated at higher temperature (29°C). Intestines were dissected at the indicated age and phosphorylated Histone H3 was detected by immunohistochemistry. B. The size of GFP+ cell clusters can be used to evaluate dysplasia in esgGal4, UAS-GFP flies (see also Text S1). The 4 categories defined visually in the panels on the left correlate with the frequency of pH3+ cells in the gut (right). C. Activation of JNK and IIS pathways in ISC (esg>Hep and esg>inR respectively) induces over-proliferation as early as 5 days, while inhibition of JNK (esg>Bsk^RNAi^) prevents tissue regeneration as shown by much reduced frequency of pH3+ cells. The TARGET system was used to prevent developmental effects of esg-driven transgenes expression (Genotypes: *w* ^1118^;esgGal4,UAS-GFP/+;tubGal80^ts^, *w* ^1118^;esgGal4,UAS-GFP/UAS-Hep^WT^;tG80^ts^, *w* ^1118^;esgGal4,UAS-GFP/UAS-InR^WT^;tG80^ts^, and *w* ^1118^;esgGal4,UAS-GFP/UAS-Bsk^RNAi^;tG80^ts^). Flies were reared at 18°C and then aged at 29°C to restrict expression of transgenes to adulthood. Averages and SEM are shown. * p<0.001 compared to Control at 5 days; # p<0.001 compared to Control at 18 days using Student's t-test. D. Intestinal dysplasia in the flies described above was monitored using the method described in Figure 1B (see also Text S1) after 18 days. Activation of JNK and IIS pathways causes accelerated dysplasia, reduction of JNK signaling leads to a complete prevention of tissue regeneration. p-value from Pearson XiSquare test. E. Flies with impaired intestinal homeostasis and tissue regeneration are short-lived. The mortality of the flies described above was recorded at 29°C. Detailed lifespan analysis is shown in Table S1.

We next assessed the relationship between ISC proliferation rates, intestinal dysplasia and lifespan (Figure 1). In wild-type flies, the number of dividing ISCs detectable at a given timepoint (as measured by the number of pH3^+^, phosphorylated Histone H3 positive, cells in the gut) increases 10-fold between 3 days and 30 days of age when reared at 25°C or between 3 days and 18 days of age when reared at 29°C (Figure 1A, [42]). This increase is accompanied by a progressive accumulation of polyploid, mis-differentiated cells that accumulate at the basal membrane of the epithelium and can be visualized by their continuous expression of the ISC/EB marker escargot (esg; [42], [43], [54]). This dysplastic phenotype is readily observed in old flies expressing GFP under the control of the esgGal4 driver, and can be classified into four distinct categories that correlate with the frequency of pH3^+^ cells per gut and thus serve as an accessible quantitative criterion for intestinal dysplasia within a fly population (Figure 1B and Text S1). Importantly, age-related dysplasia is not accompanied by aberrant proliferation of ISC daughter cells, as confirmed by analysis of pH3^+^ cell frequencies and clonal growth rates in individual marked ISC lineages in old flies or in stress conditions (Figure S3). This is consistent with previous ISC lineage analysis demonstrating that no transit amplifying population of cells exist in the *Drosophila* midgut [48]. The frequency of pH3^+^ cell numbers in the gut is thus a direct measure of ISC proliferation rates.

To influence intestinal proliferation rates in aging flies, we modulated the activities of the JNK or IIS pathways in ISCs and EBs using the esgGal4 driver. Activation of both pathways with this driver increases ISC proliferation [43], [53], [54]. We activated or inhibited JNK by expressing the JNK Kinase Hemipterous (Hep) or dsRNA against the JNK Bsk (Bsk^RNAi^), respectively, or activated IIS by expressing the Insulin receptor (InR). We used the heat-inducible esgGal4, tubGal80^ts^ system to prevent expression of transgenes before adulthood, and therefore assessed age-related changes in ISC proliferation and intestinal homeostasis at 18 days of age at 29°C (Figure 1C and 1D). Consistent with previous findings, activating JNK or insulin signaling activity resulted in dramatically increased dysplasia at 18 days and elevated proliferation rates even in young flies (5 days of induction), while when JNK signaling was impaired in the ISC lineage, dysplasia was almost entirely prevented and ISC proliferation was strongly impaired.

Interestingly, both of these extreme conditions, accelerated dysplasia and strongly inhibited ISC proliferation, resulted in significant lifespan shortening, supporting our hypothesis that intestinal homeostasis and the regenerative capacity of the intestinal epithelium are critical for fly lifespan (Figure 1E, Table S1). Maintaining intestinal proliferation rates at levels that preserve regenerative capacity while limiting dysplasia might thus influence lifespan positively.

### Reduced age-associated dysplasia in long-lived IIS loss-of-function conditions

**Figure 2:**
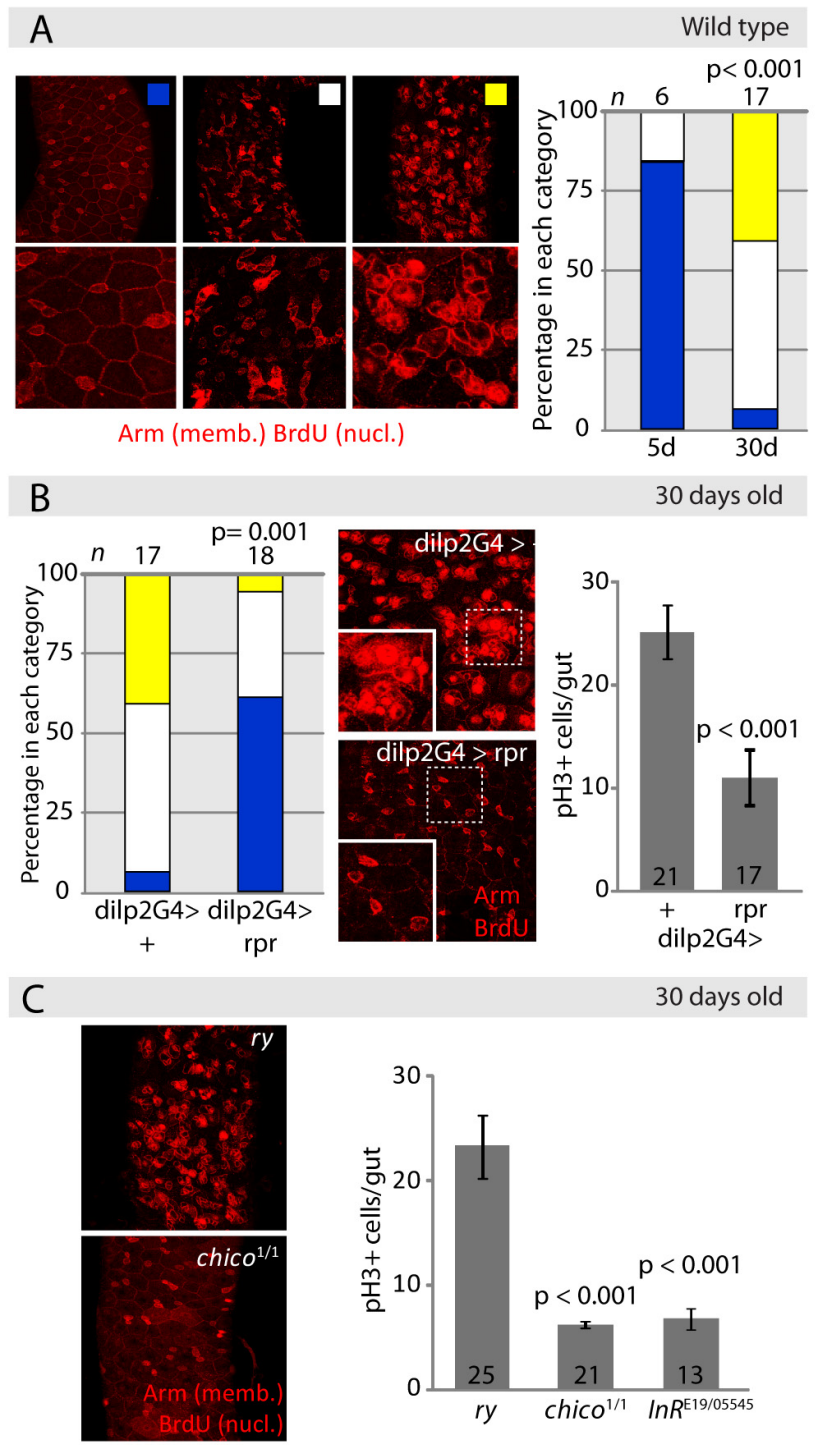
Reduced IIS activity delays tissue degeneration in the intestine. A. Evaluation of intestinal dysplasia in aging wild-type flies. BrdU incorporation identifies proliferating cells (nuclear, red), while immunohistochemistry with anti-Armadillo antibodies detects changes in epithelial structure (membrane, red). Flies were aged at 25°C, and fed BrdU for 48 hrs. B. Reducing systemic insulin signaling by ablation of Insulin Producing Cells (IPCs) delays aging-associated dysplasia. Representative pictures of the midgut from aging (30 days old at 25°C) control flies (*w* ^1118^;dilp2Gal4>+) and flies with ablated IPC's (*w* ^1118^;dilp2Gal4>rpr) are shown in the center. Scoring was performed based on the classification shown in B. Significant delay of dysplasia in dilp2Gal4>rpr can be observed compared to dilp2G>+ controls (left panel, Pearson Xi Square test). This correlates with reduced numbers of pH3^+^ cells in dilp2Gal4>rpr (Average and SEM; Student's t-test). C. Frequency of pH3^+^ cells in aging *chico^1/1^* homozygotes and *Inr^E19/05545^* compared to isogenic wild-type controls (*ry^506^*) (Average and SEM, Student's t- test). Representative BrdU/Armadillo-stained midguts from aging *chico^1^* mutant flies and sibling controls (*ry*) are shown on the left.

To test this idea, we first evaluated proliferative homeostasis in long-lived fly populations (Figure 2). Intestinal dysplasia in non-labeled intestines can be quantified by defining categories based on the extent of BrdU incorporation in the intestinal epithelium (reflecting both ISC divisions and endoreplication of daughter cells), and the loss of tissue architecture observed when staining with the membrane marker *armadillo* (*arm*, Figure 2A, see Text S1). Since reduced IIS activity extends lifespan in flies, we assessed whether long-lived fly lines with reduced IIS activity would exhibit delayed dysplasia. Indeed, limiting IIS activity systemically reduces the age-associated increase in the frequency of pH3^+^ cells, as well as the increase in intestinal BrdU incorporation and the loss of epithelial architecture in the gut (Figure 2B and 2C). IIS activity was reduced by ablating insulin-producing cells (IPCs) through expression of the pro-apoptotic gene *reaper* (*rpr*) under the control of dilp2Gal4, by reducing the genedose of the insulin receptor substrate-homologue Chico, or in trans-heterozygotes for the insulin receptor loss-of-function alleles *InR^E19^* and *InR^05545^*. Flies with all three genetic conditions are robustly long-lived [56]–[59], suggesting that the reduction in intestinal dysplasia observed here is associated with longevity.

Interestingly, in these long-lived lines, IIS activity is reduced, but not absent, since the insulin receptor can signal directly to PI3K, bypassing the requirement for Chico [60], and ablation of IPCs results in loss of selected insulin-like peptides, whereas insulin-like peptides expressed in other tissues, such as the fatbody and germline, are retained [61]–[64]. Accordingly, the average number of pH3^+^ cells decreased significantly, but moderately, indicating that in these long-lived animals, proliferative homeostasis is preserved without negatively impacting regenerative capacity (Figure 2C and 2D).

### Repression of IIS in the ISC lineage inhibits ISC proliferation and shortens lifespan

**Figure 3:**
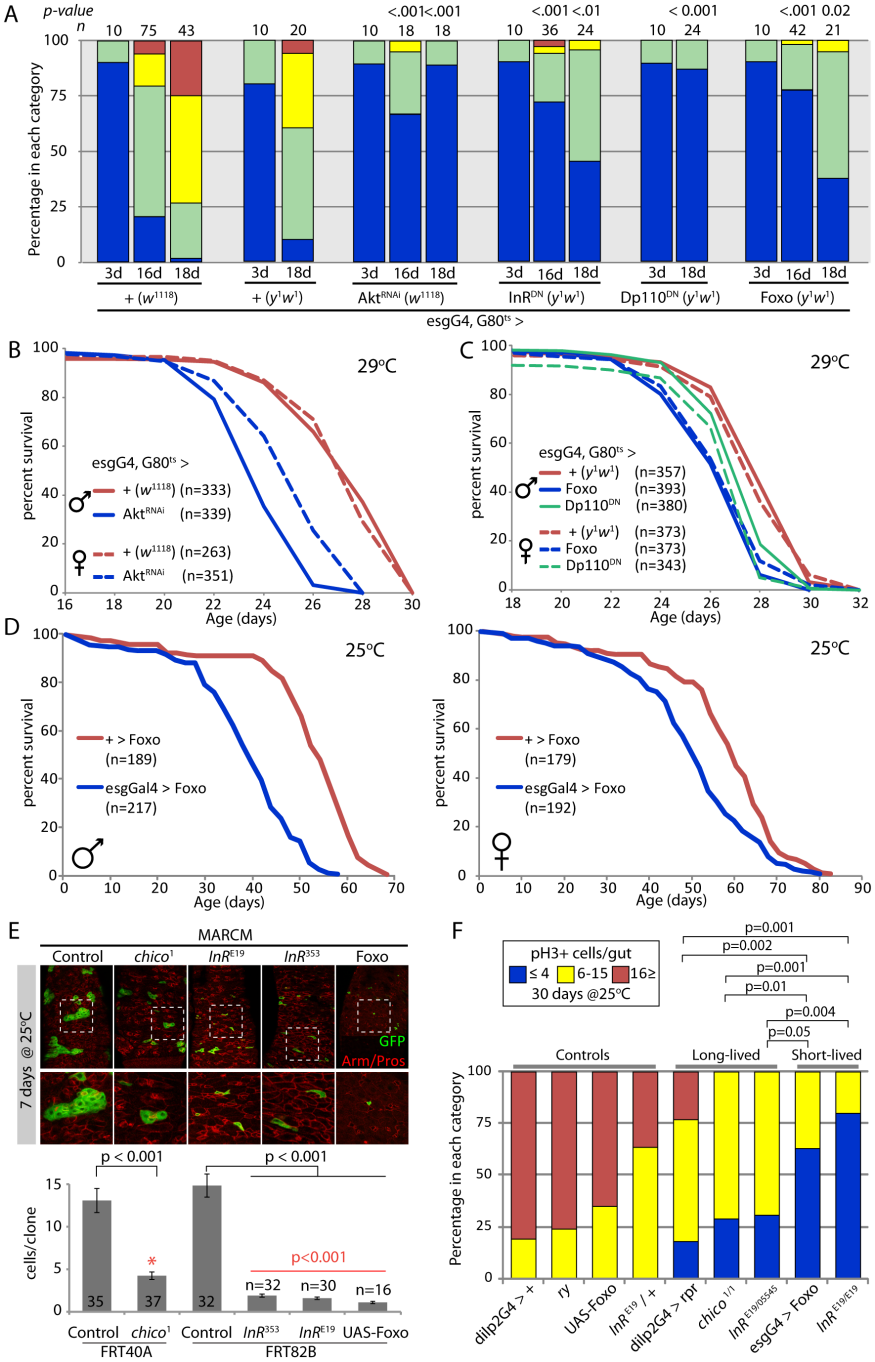
Strong reduction of insulin signaling in the somatic stem cell lineages delays age-related dysplasia and shortens lifespan. A. Intestinal degeneration was monitored in aging control flies (*w* ^1118^;esgGal4,GFP;Gal80^ts^ and *y* ^1^ *w* ^1^;esgGal4,GFP;Gal80^ts^) and flies with impaired insulin signaling activity in ISCs (*w* ^1118^;esgGal4,GFP;Gal80^ts^/UAS-Akt^RNAi^, *y* ^1^ *w* ^1^;esgGal4,GFP;Gal80^ts^/UAS-Dp110^DN^, *y* ^1^ *w* ^1^;esgGal4,GFP;Gal80^ts^/UAS-InR^DN^, *y* ^1^ *w* ^1^;esgGal4,GFP;Gal80^ts^/UAS-Dp110^DN^, *y* ^1^ *w* ^1^;esgGal4,GFP/UAS-Foxo;Gal80^ts^). Strong inhibition of IIS in ISC prevents age-related intestinal dysplasia. p-value from Pearson XiSquare test. B,C. Flies with impaired intestinal homeostasis and tissue regeneration are short-lived. The mortality of the flies described above was recorded at 29°C. Detailed lifespan analysis is shown in Table S2. D. EsgGal4 was used to express Foxo in the ISC lineage. Fly lines were backcrossed into the w1118 background (10 generations) and sibling populations derived from crosses of *w* ^1118^;esgGal4/+ females with *y* ^1^ *w* ^1^;UAS-Foxo/UAS-Foxo males were compared. Flies were reared at 18°C to minimize driver activity during development, and adults were maintained at 25°C. Lifespan is significantly shortened in flies expressing Foxo under the control of esgGal4. A detailed analysis of the mortality, as well as the mortality of isogenic controls (*w* ^1118^/*y* ^1^ *w* ^1^), flies is shown in Table S3. E. Growth of *InR* and *chico^1^* homozygous mutant ISC clones and clones over-expressing Foxo in the intestinal epithelium. Clones were induced using the MARCM system by heat-shock at three days of age and clone size was evaluated at 7 days after heat shock. *chico^1^* clones exhibit reduced, but not absent growth, while *InR* mutant clones and Foxo over-expressing clones remain mostly single cells. Representative images are shown in top panels (Green: GFP; Red: Armadillo/Prospero). Clone size quantification is shown in lower graphic (Averages and SEM). p-values from Student's t-test. InR homozygous mutant clones and clones over-expressing Foxo grow significantly less than *chico^1^* mutant clones (p-values in red). F. Comparison of the proliferation rate in the intestine of controls, long-lived and short-lived flies, after 30 days at 25°C, suggesting that long-lived mutants achieve the proper balance between tissue dysplasia and absence of regeneration. p-value from Pearson XiSquare test.

The Insulin signaling pathway has wide-ranging functions in growth, metabolism, and reproduction [6]–[8], [65]–[68]. The observed correlation between the extent of age-related dysplasia and lifespan in the genetic conditions tested above could thus be a secondary consequence of other physiological changes. To test more directly whether impairing IIS activity in the ISC lineage would influence age-related dysplasia and affect lifespan, we over-expressed a dominant-negative Insulin receptor (InR^DN^; [69], dominant-negative PI3Kinase (DP110^DN^ [70]), a dsRNA targeting the IIS downstream kinase Akt (Akt^RNAi^, Figure S4), as well as wild-type Foxo under the control of heat-inducible esgGal4 (esgGal4, tubGal80^ts^). In all four cases, we observed strongly reduced age-related dysplasia of the intestinal epithelium, confirming that IIS activity in ISCs is required for the age-related over-proliferation of these cells (Figure 3A). However, this reduction was as strong as when JNK was repressed by expression of Bsk^RNAi^, suggesting that impaired regeneration in these guts might also limit viability and reduce lifespan. We tested this prediction and found indeed that expression of Foxo, Akt^RNAi^, or DP110^DN^ under the control of esgGal4, Gal80^ts^ (at 29°C) or of Foxo under the control of esgGal4 (25°C) caused significant lifespan shortening (Figure 3B–3D, Figure S5, Table S2, Table S3).

Reduced IIS activity in the ISC lineage thus shortens lifespan most likely by preventing regeneration. Supporting this interpretation, we found that ISC clones (induced by somatic recombination using the MARCM system; [71], [72] homozygous for *InR^E19^* or *InR^353^*, or over-expressing Foxo, have a strongly reduced ability to grow (and thus to generate newly differentiated ECs and EEs, Figure 3E). The extent of this growth repression was significantly more severe than in *chico^1^* homozygous mutant clones (Figure 3E). Interestingly, *InR^E19^* homozygous mutant flies are short-lived (as opposed to *InR^E19^/InR^05545^* transheterozygotes; [56]), further strengthening the notion that impaired regeneration of the intestinal epithelium of these flies is associated with shorter lifespan.

### Lifespan extension by limiting IIS and JNK signaling in somatic stem cells

Taken together, the results described above support the notion of a critical relationship between proliferative homeostasis, regeneration and lifespan: reduced ISC proliferation, and thus limited age-related dysplasia (as in dilp>rpr, *chico^1^* homozygotes and InR^E19^/InR^5545^ transheterozygotes), is beneficial, while impaired ISC proliferation, and thus reduced regenerative capacity (as in *InR^E19^* homozygotes or in flies over-expressing Foxo in the ISC lineage), shortens lifespan. This relationship can be illustrated by comparing relative lifespan with the fraction of flies with low, intermediate or high frequencies of ISC proliferation at 30 days (reared at 25°C) for the genotypes discussed above (Figure 3F).

**Figure 4:**
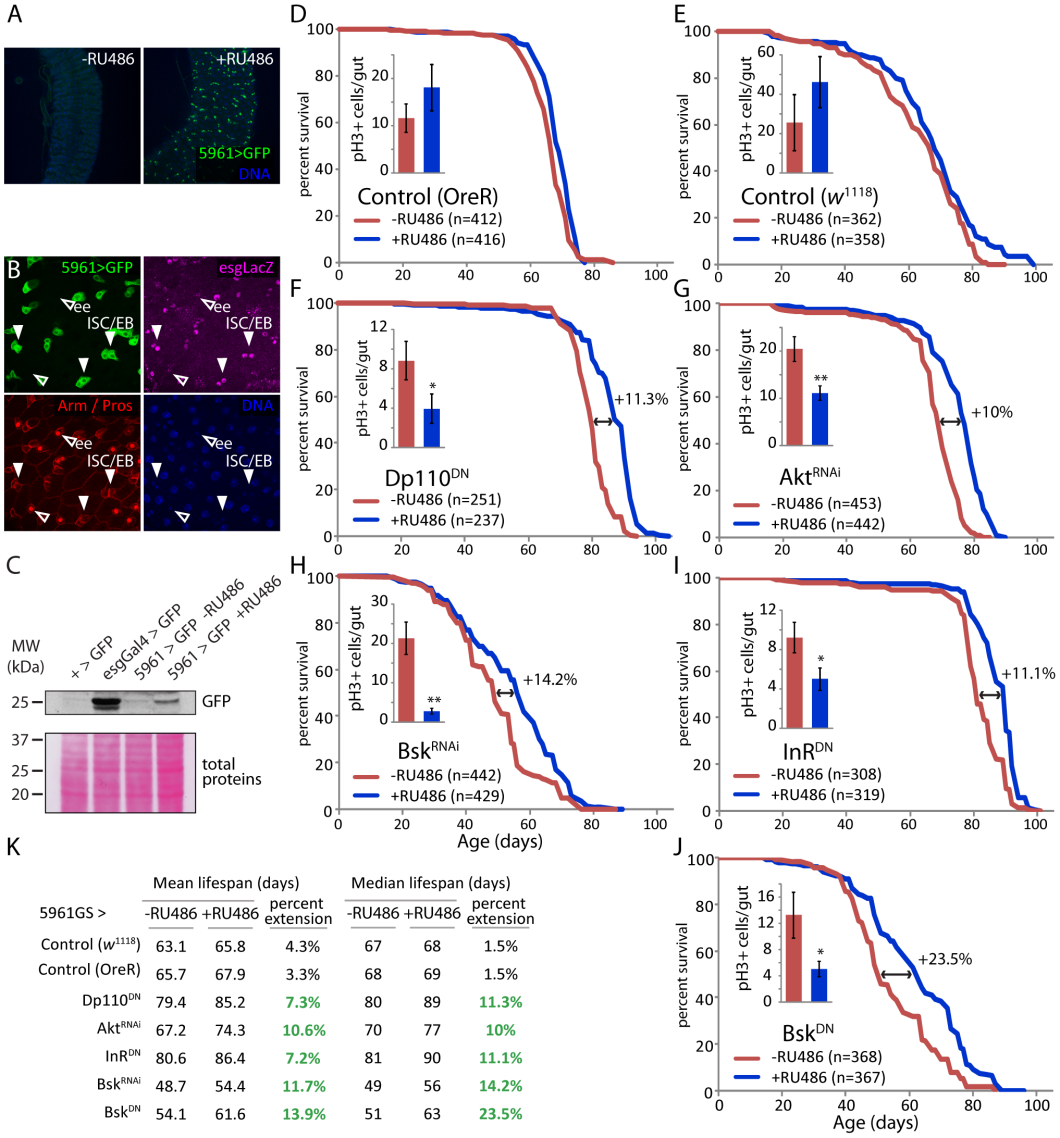
Moderate inhibition of IIS and JNK pathways in somatic stem cell lineages extends lifespan. A. The 5961GS driver is expressed in the intestine and responsive to RU486. GFP can be detected in the posterior midgut of 5961GS>GFP flies after RU486 exposure, no GFP is detected when flies are kept on control food. B. In the intestine, the activity of the 5961GS driver is restricted to ISC and EB. Only LacZ-positive cells express GFP in 5961GS>GFP/esg-LacZ flies. The expression of the reporter esg-LacZ identifies ISC and EB, immunostaining against prospero identifies EE. C. Western-blot analysis of total extract from dissected guts shows that GFP can be detected in the intestine of 5961GS>GFP flies after RU486 exposure. However, the expression level remains much lower than in the intestine of esgGal4>GFP flies. D–J. Moderate reduction of the IIS (F, G, I) and JNK (H, J) pathways using 5961GS extends lifespan. The mortality of sibling flies of the indicated genotypes placed on control food (-RU486) or food supplemented with RU486 (+RU486) was compared at 25°C. The treatment has minimal effect on the longevity of control flies (5961GS,UAS-GFP>+ in *w* ^1118^ and OreR background), but causes significant increase in longevity of flies with reduced IIS and JNK pathways (5961GS,UAS-GFP>UAS-InR^DN^, 5961GS,UAS-GFP>UAS-Dp110^DN^, 5961GS,UAS-GFP>UAS-Akt^RNAi^, 5961GS,UAS-GFP>UAS-Bsk^DN^, 5961GS,UAS-GFP>UAS-Bsk^RNAi^). The relative extension of the median lifespan is shown for each genetic condition. For each condition, the reduction of ISC proliferation by the treatment was confirmed, as measured by the number of pH3^+^ cells in the intestinal epithelium, in 50 to 70 days old females (n>12 guts; Averages and SEM; p-values from Student's t-test * p<0.05, ** p<0.01). K. Summary of lifespan statistics including mean and median lifespan (days) for all conditions. Detailed lifespan analysis is shown in Table S4.

To test this model more directly, and to confirm that improved proliferative homeostasis is sufficient to extend lifespan, we repressed IIS and JNK activities in ISCs and EBs using 5961 Geneswitch-Gal4 (5961GS; Figure 4, [73]). This RU486-inducible driver recapitulates the esgGal4 expression pattern in the intestine, albeit at much lower levels, allowing moderate repression of IIS and JNK activities in an RU486-dependent manner ([73], Figure 4A–4C; we assessed GFP expression under the control of 5961GS in various tissues, and observed weak RU486-dependent induction only in the intestine, Figure S6). Female flies expressing InR^DN^, DP110^DN^, Akt^RNAi^, Bsk^DN^, or Bsk^RNAi^ under the control of this driver show moderately, but significantly, reduced intestinal proliferation at old age (Figure 4D–4J). Importantly, these flies are significantly longer lived when exposed to RU486 than isogenic siblings exposed to mock treatment (median lifespan extended at least 10% for all conditions); whereas control flies show almost no RU486-dependent change in longevity (1% change in median lifespan; Figure 4D–4K and Table S4; expression of the same transgenes in males resulted in no significant lifespan effect, not shown). All together, these results strongly support the model outlined above.

### Expression of stress-protective genes in the ISC lineage limits age-associated dysplasia

IIS and JNK signaling activities thus have to be carefully balanced to maintain intestinal homeostasis and regenerative capacity. This balance will ultimately influence the expression of Foxo target genes, which encode stress-protective proteins as well as cell cycle inhibitors and pro-apoptotic factors that are expected to have antagonistic consequences for stem and progenitor cell maintenance and proliferation. Accordingly, a critical and pleiotropic function of Foxo proteins in stress-protection, proliferation and apoptotic control of stem cells has been described for the hematopoietic system in mice [18], [29]–[32]. Selectively increasing the expression of stress-protective Foxo target genes in the ISC lineage might thus be sufficient to limit age-related dysplasia without impairing regeneration, thus recapitulating the consequences of organism-wide moderate reduction of IIS activity, and potentially extending lifespan.

**Figure 5:**
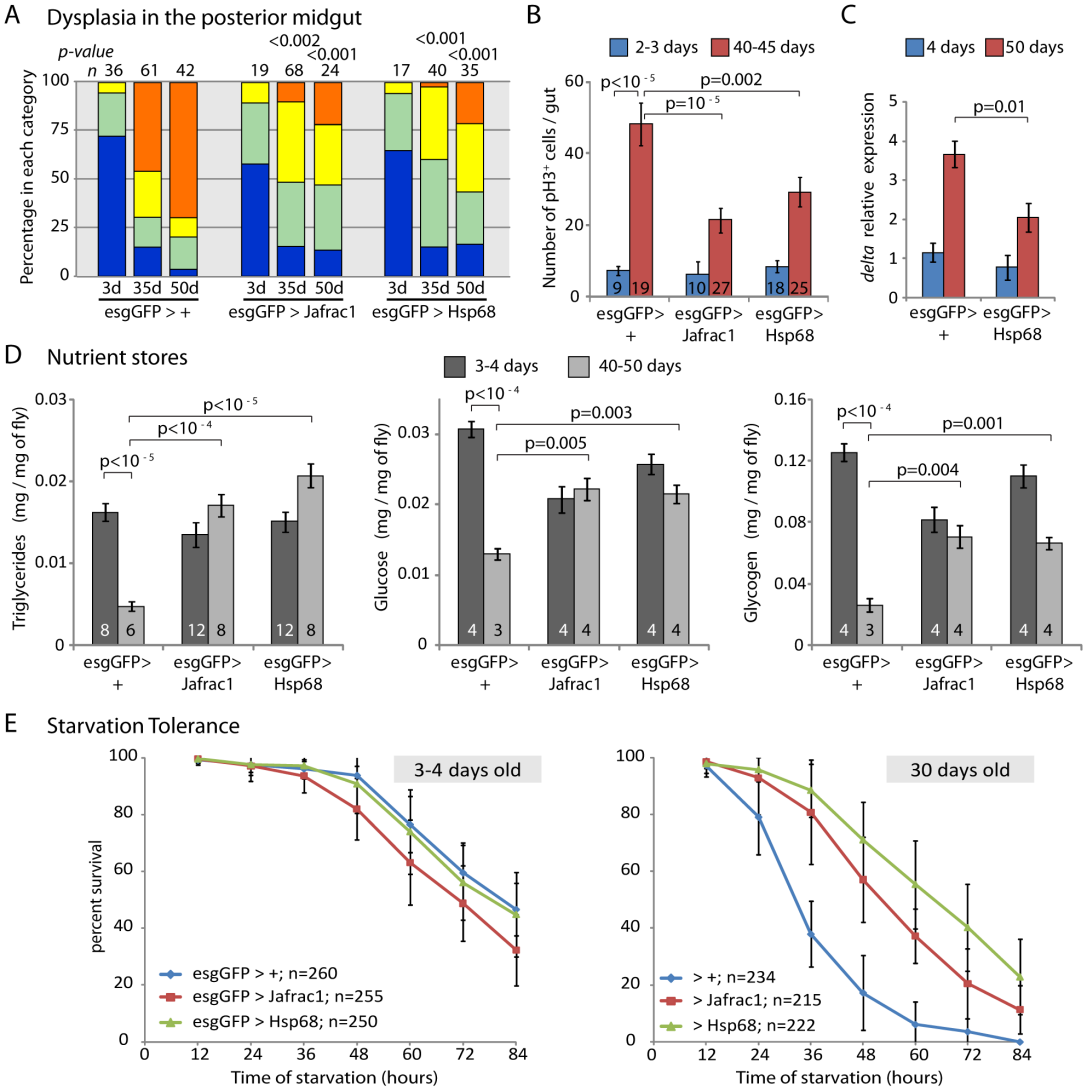
Overexpression of stress-protective genes in the somatic stem cell lineages delays intestinal degeneration and limits metabolic decay. A. Overexpression of Jafrac1 or Hsp68 delays age-related loss of intestinal architecture. Intestinal degeneration in aging (3, 35, and 50 days) control flies (esgGFP>+) and flies overexpressing cytoprotective genes in the ISCs (esgGFP>Jafrac1 and esgGFP>Hsp68) was scored in the posterior midgut. p- values from Pearson Xi Square test. B. Overexpression of Jafrac1 or Hsp68 under the control of esgGal4 also limits the increase in the frequency of pH3^+^ cells in aging intestines (Averages and SEM; Student's t-test). C. *Dl* expression relative to *rp49* in the aging intestine measured by real-time RT-PCR (Averages and SEM; Student's t-test). D. Over-expression of Jafrac1 or Hsp68 delays age-related changes in nutrient levels. Triglycerides, free glucose and glycogens were measured in young (3–4 days old) or old (40–50 days old) flies. Concentration is shown as mg nutrient per mg fresh fly. The number at the bottom of each bar represents the number of samples. All error bars represent standard deviation, p-value from Student's t-test. E. Jafrac1 and Hsp68 expression in ISCs increases starvation tolerance in old flies. Wet starvation resistance was determined in the indicated populations of flies aged for three days (left) or for 30 days (right).

To test this hypothesis, we used esgGal4 to express Hsp68, a heatshock protein that extends lifespan when expressed in the whole fly [74], and Jafrac1, a peroxiredoxin that detoxifies ROS and can increase lifespan when expressed in the brain [75], [76]. Both genes are Foxo targets (Figure S7; [75]) and strikingly, we found that both caused a significant delay in dysplasia (both in the posterior midgut, as well as when assessing invasion of the proventriculus by GFP-positive cells; Figure 5A and Figure S8), accompanied by moderate reduction in the frequency of pH3^+^ cells in the gut (Figure 5B). Since dysplasia in aging intestinal epithelia is accompanied by increased expression of *Dl* [43], we further tested the expression of *Dl* in these intestines and found a significant decrease in *Dl* accumulation compared to wild-type animals, confirming that the accumulation of mis-differentiated ISC progeny in these flies is reduced (Figure 5C).

### Improved metabolic homeostasis in flies expressing stress-protective genes in somatic stem cell lineages

Dysplasia in the aging intestinal epithelium is expected to cause defects in nutrient absorption, resulting in deficient nutrient stores in the organism and disrupting metabolic homeostasis. Since *hsp68* and *jafrac1* expression in ISCs and their daughter cells significantly delays intestinal dysplasia, we tested whether the maintenance of metabolic homeostasis was improved in these flies. The amount of free glucose, triglycerides and glycogen stored by old wild-type flies is significantly reduced compared to young animals (Figure 5D). When *jafrac1* or *hsp68* were expressed under the control of esgGal4, however, high levels of these nutrient stores were maintained in aging flies. This rescue of metabolic homeostasis correlates with increased starvation tolerance (Figure 5E), further supporting the idea that maintenance of intestinal homeostasis by protecting somatic stem cells is critical for metabolic health of aging flies.

#### Lifespan extension by stress-protective gene expression in stem cell lineages

**Figure 6:**
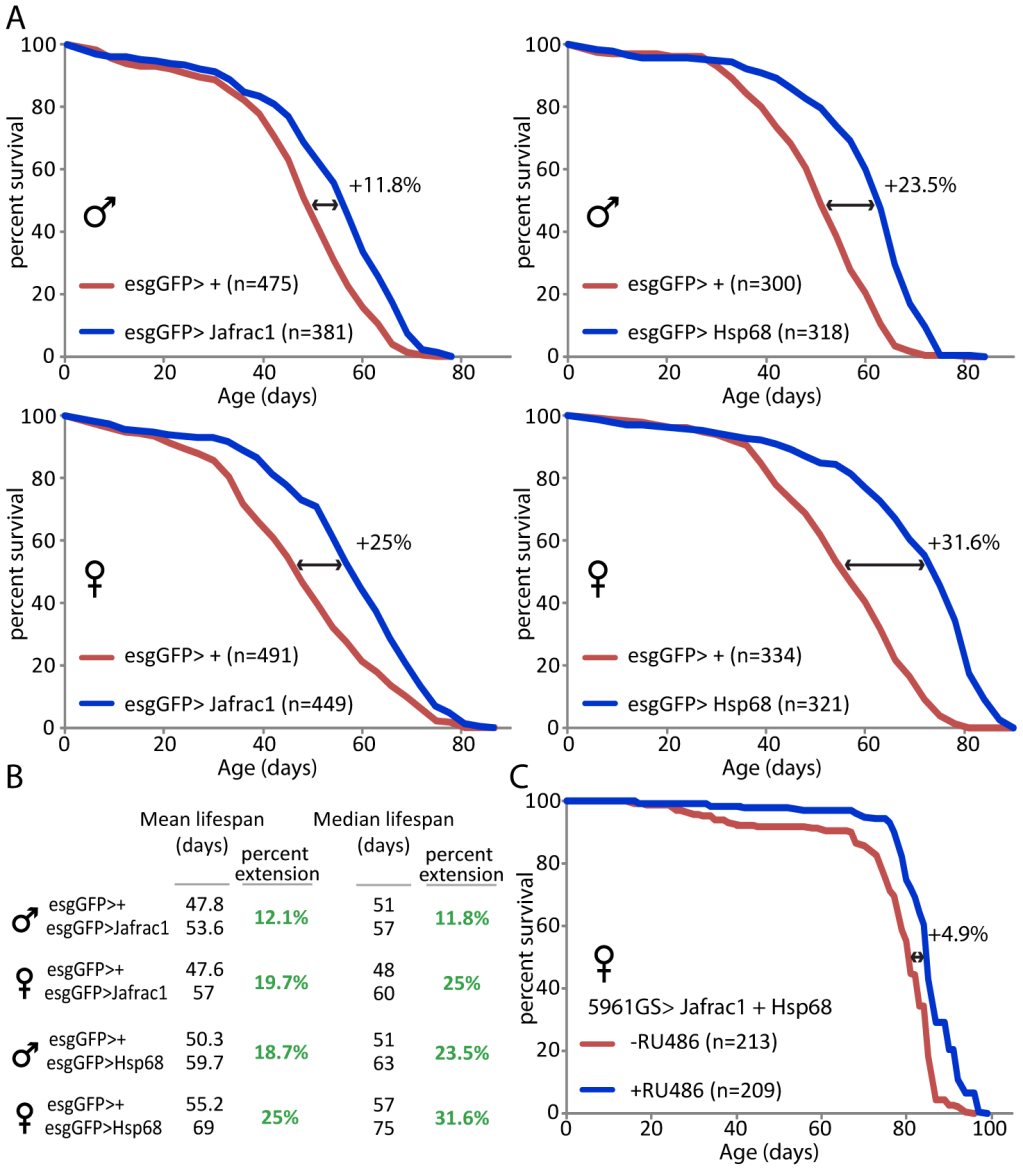
Over-expression of stress-protective genes in the somatic stem cell lineages extends lifespan. A. Survival curves of esgGFP>Jafrac1 and esgGFP>Hsp68 flies compared to their respective wild type isogenic controls. UAS lines were backcrossed 10 generation into *w* ^1118^ background. Wild type and UAS siblings were crossed to esgGFP and mortality of the progeny was recorded at 25°C. B. Summary of lifespan statistics including mean and median lifespan (days). C. Over-expression of Jafrac1 and Hsp68 using the 5961GS driver moderately extends lifespan. The mortality of sibling flies (5961GS,GFP>UAS-Jafrac1,UAS-Hsp68) placed on control food or food supplemented with RU486 was compared at 25°C. Due to the weak activity of the 5961GS driver, both UAS-Jafrac1 and UAS-Hsp68 transgenes were combined to observe a significant effect. The relative extension of the median lifespan is shown for all curves. Detailed lifespan analysis is shown in Table S5 and S6.

To assess whether over-expressing stress-protective genes using the esg-Gal4 driver would be sufficient to extend lifespan, we compared demographies of multiple independent populations of flies expressing *jafrac1* and *hsp68* under the control of esgGal4 to isogenic wild-type controls. Strikingly, we observed consistent and significant lifespan extension in both males and females when *jafrac1* and *hsp68* were expressed (Figure 6A and 6B; Table S5). To exclude that the esgGal4-driven expression of stress-protective genes in salivary glands is causing the observed lifespan extension, we also tested the lifespan of flies in which hsp68 was expressed using GMR-Gal4, an eye-specific driver that also expresses Gal4 in salivary glands, and found no effect (Figures S1B, S1C, S9). We further confirmed the beneficial consequences of Jafrac1 and Hsp68 expression on lifespan using the weaker 5961GS driver, and found moderate but significant extension of lifespan in flies expressing both transgenes (Figure 6C, Table S6). Evidently, expressing selected stress-protective Foxo target genes in the ISC lineage is sufficient to recapitulate the effects of reducing IIS or JNK activity in these cells.

## Discussion

**Figure 7:**
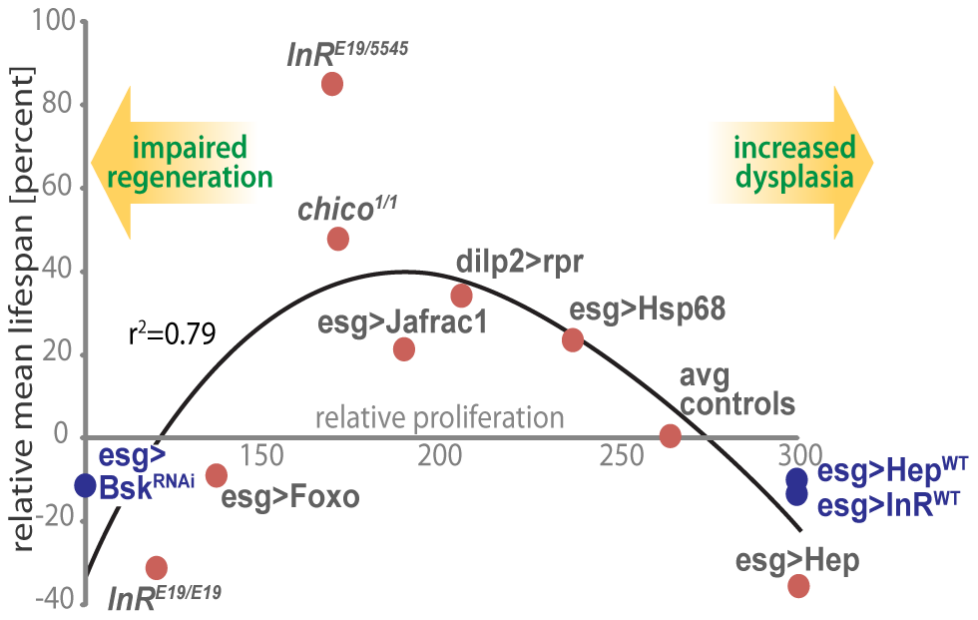
A model for the impact of regenerative capacity on lifespan. Genetic conditions that moderately decrease ISC proliferation (thus limiting dysplasia) are associated with increased lifespan, while strong repression of ISC proliferation is deleterious for regeneration and shortens lifespan. The association of lifespan and regenerative capacity of the intestine in aging flies is illustrated by comparing ISC proliferation rates and lifespan. Relative ISC proliferation rates were calculated for each genotype using the three categories defined in Figure 3F (low, intermediate and high frequencies of pH3+ cells) and the following formula: ×  =  (proportion in cat.1) + 2*(proportion in cat.2) + 3*(proportion in cat.3). This model includes lifespan and intestinal proliferation analysis from experiments conducted at 25°C (red dots) or 29°C (blue dots). The relative proliferation data is from this study. These data are plotted against lifespan changes in the respective genotypes relative to corresponding isogenic controls, from this work and from published studies [56], [57], [59]. Polynomial regression curve (3^rd^ degree) was fitted using Excel.

Our results indicate that proliferative homeostasis in high turnover tissues is limiting for *Drosophila* lifespan and highlight the importance of mechanisms that balance pro- and anti-mitotic activities. In the ISC lineage, this balance involves fine-tuning the activities of the pro-mitotic IIS and JNK signaling pathways to ensure appropriate supply of newly formed ISC daughter cells while limiting dysplasia. Accordingly, we observe moderately reduced intestinal proliferation rates in long-lived IIS mutants, as well as lifespan extension when IIS or JNK signaling are moderately reduced in the ISC lineage. This association between proliferative activity in the intestinal epithelium and lifespan is illustrated in Figure 7. Strikingly, intestinal proliferation rates correlate with relative lifespan over a wide range of genotypes.

Our results further show that the stress-protective components of the Foxo-regulated gene expression program are sufficient to maintain proliferative homeostasis, extending lifespan of the organism. Reduction of IIS activity, which extends lifespan in many organisms, is thus accompanied by the preservation of regenerative processes. While reducing IIS activity or activating Foxo in adipose tissue is sufficient to extend lifespan of flies, mice and worms [65]–[68], our results suggest that the anti-proliferative and stress-protective consequences of Foxo activation in high-turnover tissues also contribute to lifespan extension in IIS loss-of-function conditions. Interestingly, a tumor-suppressing role for Foxo in mice and *C.elegans* has been reported [77]–[79], while Foxo regulates redox homeostasis in mouse HSCs [18], [29], [30], [32], [77]. Reduced IIS activity thus optimizes somatic maintenance, metabolism and regenerative processes in complex metazoans, and all three physiologic consequences of IIS repression seem to contribute to achieve maximum lifespan.

It remains to be tested whether the lifespan extension commonly observed in flies exposed to dietary restriction (DR) is also associated with delayed intestinal dysplasia. Reduced IIS activity contributes to lifespan extension in DR conditions [80]–[82] suggesting that reduced intestinal dysplasia might contribute to DR-induced lifespan extension.

Interestingly, the effects of JNK signaling on lifespan are more complex. JNK can extend lifespan when activated in the brain by repressing the expression of insulin-like peptides [37], [74], [83], [84], thus systemically repressing IIS activity [37], [74]. Our findings reported here, however, show that JNK activation in the ISC lineage can have deleterious effects and needs to be limited to ensure longevity. Such pleiotropic consequences of JNK have also been reported in other contexts and have significant implications for the development of therapies targeting this pathway [85]–[87].

The importance of anti-oxidant Foxo target genes in regulating proliferative homeostasis highlights the challenging environment to which the intestinal epithelium is exposed. Apart from extraneous toxins and oxidants, the intestinal epithelium also mounts strong oxidative responses to inflammation, potentially exposing ISCs and daughter cells to high levels of oxidative stress [50], [52], [88]–[91]. Our results show that over-expressing stress-protective proteins in the ISC lineage is sufficient to limit and optimize cellular responses to these challenges, thus preserving intestinal homeostasis longer (but not indefinitely, as a significant fraction of these animals do develop dysplasia at older ages).

Intestinal dysplasia is caused by over-proliferation of ISCs in concert with mis-differentiation of ISC progeny, and in long-lived animals both processes are prevented. Due to technical limitations of the Gal4 drivers used, however, we cannot exclude that the expression of stress-protective genes, or of IIS or JNK repressors with esgGal4 or 5961GS affects primarily the differentiation process of EBs rather than the ISC itself. While the inability of IIS mutant clones to grow, and the significant reduction in the number of pH3^+^ cells in IIS and JNK loss-of-function conditions and in Hsp68 and Jafrac1 over-expressing flies, demonstrates that ISC proliferation is indeed influenced by these manipulations, it is conceivable that this effect might be mediated by indirect, non-cell-autonomous limitation of ISC proliferation by EBs in these conditions. Such a feedback control of ISC division would be interesting, and further studies are needed to test this possibility.

The pattern of esgGal4 and of 5961GS expression further requires considering effects of IIS and JNK activities in other tissues on lifespan: While we can exclude the testes and salivary glands as sources of the observed effects (lifespan effects are observed in both males and females using esgGal4, expression of Jafrac1 and Hsp68 in salivary glands has no effect on lifespan, and 5961GS is not expressed in salivary glands), we cannot currently exclude a contribution of malpighian tubule stem cells. These cells also appear to respond to proliferative signals such as Hep or InR over-expression, and over-proliferate in stressed flies (GFP-labeled cells accumulate in malpighian tubules in these flies; Biteau, unpublished), but the exact mechanism of regeneration and a potential age-related dysplastic phenotype in this tissue remain unexplored. Importantly, a contribution of this somatic stem cell population to the lifespan effects reported here would further support our model of the importance of proliferative homeostasis in high-turnover tissues for *Drosophila* lifespan.

It is interesting that using the weaker 5961GS driver, lifespan extension in IIS and JNK loss-of-function conditions is only observed in females. This sexual dimorphism might be a consequence of a slight difference in driver activity between the sexes (no significant difference in driver activity can be observed, however), or might indicate selective sensitivity of females to intestinal dysplasia. Interestingly, intestinal turnover rates in females are higher than in males [50], indicating a potential reason for such a selective sensitivity. Accordingly, lifespan extension by esg-mediated expression of Hsp68 and Jafrac1 is also stronger in females than in males.

Based on the highly conserved regulation of regenerative processes in flies and vertebrates [13], [14], [25], [47]–[49], our findings suggest that interventions that focus on maintaining regenerative capacity by improving stem and progenitor cell stress-protection hold significant promise for slowing aging in higher organisms, including humans. Interestingly, vertebrates seem to have evolved more efficient and extensive cell autonomous anti-proliferative mechanisms in stem cells than flies [20]–[22], resulting in longer-lasting maintenance of homeostasis in high-turnover tissues. The rapid decay of intestinal homeostasis in flies indicates that such control mechanisms have not been acquired in these short-lived animals, yet our data also suggest the potential for active control of proliferation rates in the intestinal epithelium by systemic insulin-like peptide levels. Interestingly, the regulation of stem cell proliferation by IIS and Foxo is conserved in mammalian systems, suggesting that similar systemic control of stem cell proliferation could be harnessed to regulate regenerative capacity and lifespan in vertebrates [18], [31]. How the maintenance of intestinal homeostasis is influenced by environmental parameters that affect systemic IIS activity is an interesting subject of further studies.

## Materials and Methods

### Drosophila stocks and culture

The following strains were obtained from the Bloomington Drosophila Stock Center: *w^1118^*, *ry* ^506^, *y* ^1^ *w* ^1^, UAS-InR^DN^, UAS-Dp110^DN^, UAS-rpr, and tub-Gal80^ts^. UAS-Akt^RNAi^ and UAS-Bsk^RNAi^ were obtained from the Vienna Drosophila RNAi Center (transformant ID 2902 and 34138). esg-Gal4 was kindly provided by S. Hayashi; *chico* ^1^ and UAS-FoxoTM by M. Tatar; dilp2-Gal4 by E.Rulifson; UAS-Hep and sep-Gal4 by M. Mlodzik; UAS-NICD by N.Perrimon. MARCM stocks were gifts from N. Perrimon (hsFlp; tub-Gal4,UAS-GFP;FRT82B tubGal80) and B.Ohlstein (hsFlp; FRT40A tub-Gal80; tub-Gal4,UAS-GFP). 5961GS was a gift from B.Ohlstein. FRT chromosomes were kindly provided by D. Drummond-Barbosa (FRT40A *chico* ^1^, FRT82B *InR* ^E19^ and FRT82B *InR* ^353^). The UAS-Foxo and UAS-Hsp68 were described previously [37], [74]. The UAS-Jafrac1 transgene was constructed by cloning the coding sequence of the *jafrac1* gene, amplified from cDNA by using the following primers: 5′-ATGCCCCAGCTACAGAAGC-3′ and 5′-TTAGGAGGTGGTCTCGAAG-3′, into a pUAST vector. Transgenic flies were generated using standard procedures.

All flies were raised on the following food: 1 liter distilled water, 13.8 g agar, 22 g molasses, 80 g malt extract, 18 g Brewer's yeast, 80 g corn flour, 10 g soy flour, 6.25 mL propionic acid, 2 g methyl-p-benzoate, 7.2 mL of Nipagin (20% in EtOH). Flies were kept at 25°C and 65% humidity, on a 12 h light/dark cycle, unless otherwise indicated.

### Conditional expression of UAS-linked transgenes

The TARGET system was used to conditionally express UAS-linked transgenes in ISCs [55]; the esg-Gal4 driver was combined with a ubiquitously expressed temperature-sensitive Gal80 inhibitor (esg-Gal4;tub-Gal80^ts^). Crosses and flies were kept at room temperature (permissive temperature), then shifted to 29°C to allow expression of the transgenes.

### Generation of marked homozygous mutant clones

*chico* and *InR* mutant clones were generated by somatic recombination using the MARCM stocks described above and FRT40A and FRT82B chromosomes carrying *chico* ^1^ and InR mutations, respectively. Using Flp/FRT-mediated somatic recombination with a repressible cell marker, MARCM allows generating homozygous mutant clones of cells that are positively marked (by GFP in this case). 2–4 days old flies were heat-shocked for 45 minutes at 37°C to induce somatic recombination. Clones resulting from mitotic recombination were observed 7 days after induction.

### Immunostaining and microscopy

Intact guts were fixed at room temperature for 45 minutes in 100 mM glutamic acid, 25 mM KCl, 20 mM MgSO_4_, 4 mM Sodium Phosphate, 1 mM MgCl_2_, 4% formaldehyde. All subsequent incubations were done in PBS, 0.5% BSA, 0.1% TritonX-100 at 4°C.

The following primary antibodies were used: mouse anti-BrdU (Becton Dickson) 1∶200; mouse anti-Prospero and anti-Armadillo (Developmental Studies Hybridoma Bank) 1∶250 and 1∶100; rabbit anti-pH3 (Upstate) 1∶1000. Fluorescent secondary antibodies were obtained from Jackson Immunoresearch. Hoechst was used to stain DNA.

Confocal images were collected using a Leica SP5 confocal system and processed using the Leica software and Adobe Photoshop.

### BrdU incorporation

Flies were cultured on standard food supplemented with BrdU (final concentration 0.2 mg/ml) for 2 days. Intact guts were fixed as previously described and DNA was denatured by incubating tissue in 3M HCl for 30 minutes. Samples were then processed for immunostaining as described above.

### Metabolite measurements

4 to 5 females (without the head) were homogenized in 150 µl of buffer (10 mM KH_2_PO_4_, 1 mM EDTA, pH 7.4). 10 µl of cleared extract was used to measure triglycerides, glucose and glycogen concentrations according to the manufacturer instructions (Triglyceride Liquicolor, Stanbio; Glucose and Starch Assay Kits, Sigma).

### Lifespan analysis

For lifespan experiments at 29°C using the TARGET system, virgin females (esgGal4, UAS-GFP; tubGal80^ts^) were crossed to the following UAS transgenes or the respective wild-type controls: UAS-Hep^wt^, UAS-Bsk^RNAi^, and UAS-Akt^RNAi^ (back-crossed at least 10 generations into *w^1118^*); UAS-Foxo^wt^ and UAS-Dp110^DN^ (in *y^1^,w^1^* background); and UAS-InR (in *w^1118^* background). Crosses were kept at room temperature. After collection and sorting (60–100 flies/cage), flies were placed at 29°C to age.

The UAS-Jafrac1 and UAS-Hsp68 transgenes were backcrossed 10 times into the *w^1118^* background and kept as an unbalanced stock. Of this stock, 10 to 15 homozygous males (+/+ or UAS/UAS) were independently crossed to 40 *yw; esgGal,UASGFP/CyO* virgins. To control for the effect of over-expressing Hsp68 in salivary glands, males (+/+ or UAS/UAS) from the same backcrossed UAS-Hsp68 stock were independently crossed to *w; GMR-Gal4,UAS-GFP* homozygous virgins. Crosses and progeny were kept at all times at 25°C.The progeny of these crosses was collected 2 days after hatching and allowed to mate in bottles for 3 days. Flies were finally separated according to their sex and genotype into cages (50–100 flies/cage).

To test the effect of Foxo over-expression using the esgGal4 driver on lifespan, the driver was backcrossed 10 times into the *w^1118^* background and kept as an unbalanced stock. 40 *w^1118^; esgGal4/+* virgins were crossed to 10–15 *yw; UAS-Foxo* homozygous males. Crosses were kept at 18°C to minimize driver expression and potential developmental defects associated with Foxo expression. The progeny of these crosses was collected 4 to 5 days after the first fly hatched. Flies were allowed to mate in bottles for 2 days at room temperature. Siblings were finally separated according to their sex and genotype into cages (20–50 flies/cage) and transferred at 25°C.

For RU486 food supplementation, 100 µl of a 5 mg/ml solution of RU486 or vehicle (ethanol 80%) were deposited on top of a food vial and dried for at least 16 hours to ensure complete evaporation, resulting in a 0.2 mg/ml concentration of RU486 in the food accessible to flies (determined using a dye control as previously described for drug treatments [92].

For all populations, plastic cages (175 ml volume, 5 cm diameter from Greiner bio-one) were used for lifespan experiments. Food, changed every 2 days, was provided in vials inserted into a foam plug (4.9 cm in diameter, 3 cm thick from Greiner bio-one), dead flies were visually identified (flies not moving, not responding to mechanical stimulation and laying on their side or back were deemed dead), and the number of dead flies was recorded. Cages were replaced after 20 days (flies were transferred into new cages without anesthesia). Survival of the different populations was analyzed using the SAS JMP7 statistical software.

The driver lines (esgGal4 and 5961) used to collect females for all lifespan studies are Wolbachia negative, the lifespan effects observed in flies with reduced JNK or IIS activity in somatic stem cells are thus Wolbachia independent.

### Analysis of GFP expression by western blot

Analysis of esgGal4, 5961GS and GMR-Gal4 expression pattern by western blot 5 females (esgGal4>UAS-GFP, 5961GS>UAS-GFP, GMRGal4>UAS-GFP or OregonR) were dissected into heads, guts, salivary glands, ovaries and the carcasses of the thorax and abdomen. Tissues were homogenized in protein sample buffer; proteins were separated by SDS-PAGE and transferred to nitrocellulose membrane using standard procedures. GFP was detected using rabbit anti-GFP antibody (Invitrogen; 1∶5000), HRP-conjugated anti-rabbit and chemi-luminescence, according to manufacturer instructions. Total proteins, detected using Ponceau staining, or Heterochromatin Protein 1 (detected by immune-staining, anti-HP1, DSHB; 1∶5000) are used as loading controls.

### Analysis of gene expression

Total RNA from 5 guts, from embryos, or from dissected 3^rd^ instar larval eye imaginal discs, was extracted using Trizol and cDNA synthesized using Superscript II (Invitrogen). Real time PCR was performed using SYBR Green, a Biorad IQ5 apparatus and the following primers pairs (Delta: 5′-TGA GCA CTT TCT CCT CGC ACA TCT-3′and 5′-AGG CTT GTA CTG CAA CCA GGA TCT-3′; Rp49: 5′-TCC TAC CAG CTT CAA GAT GAC-3′ and 5′-CAC GTT GTG CAC CAG GAA CT-3′; Akt: 5′-AAG CGT TTG GGA GGT GGA AAG GAT-3′ and 5′- TCA ACT CCA CAC TCT CTC CCG TAA-3′; Actin: 5′-CTC GCC ACT TGC GTT TAC AGT-3′ and 5′- TCC ATA TCG TCC CAG TTG GTC-3′; Jafrac1: 5′-CAA GTT GAG CGA CTA CAA GG-3′ and 5′-TCA TCG AGC ACT CCA TAG TC-3′). Data was calculated using the ΔCt method and normalized to actin levels. Results are average +/− standard deviation of at least 3 independent biological samples run in triplicate.

## Supporting Information

Figure S1 Expression pattern of the esgGal4 driver. A. All accessible tissues from flies expressing GFP under the control of the esgGal4 driver were dissected. GFP can exclusively be detected in the Intestinal Stem Cells (ISCs) in the midgut, the Renal Stem Cells (RNSCs) in the malpighian tubules, the testis and the salivary glands. No Fluorescence can be detected in the crop, the hindgut, the ovaries, the brain or the abdominal fat body. B. GMRGal4, used as a control for lifespan experiments, overlaps with esgGal4 expression in the salivary glands. C. Western-blot analysis of dissected tissues from adults in which esgGal4 or GMRGal4 drive expression of GFP. Extracts from OreR (wild-type) flies are shown as controls. Note the overlap of expression of the two drivers in salivary glands, and the exclusive expression of esg-GFP in the intestine and salivary glands. Mobility shift between the GFPs expressed in either line is due to expression of different GFP constructs in esgGal4 or GMRGal4 recombinants. Ponceau Red staining is shown as loading control.
(4.01 MB TIF)

Figure S2 ISC function is critical for normal lifespan. A. Inducing differentiation in the ISC lineage (using activated notch intra-cellular domain; esgGal4, UAS-GFP;tubGal80^ts^/UAS-NICD) results in shortening of lifespan compared to wild-type controls (esgGal4, UAS-GFP;tubGal80^ts^/+ (*y* ^1^ *w* ^1^)) in both males and females. Flies used in these experiments were reared at a permissive temperature (22 ° C, promoting activity of the Gal80 repressor), then transiently shifted to 29 ° C at 5 days of age (for 7 days) to deactivate the repressor and irreversibly impair stem cell function through over-expression of NICD. Flies were aged at 25 ° C. B. Longevity (of esgGal4, UAS-GFP; tubGal80^ts^/UAS-NICD) is minimally affected when flies are kept at a permissive temperature (22 ° C, promoting activity of the Gal80 repressor) throughout life. This suggests that the TARGET system (tubGal80^ts^) approach can inhibit developmental effects of transgenes, thus limiting the lifespan effects strictly to changes in the adult. C. Summary of lifespan statistics for all populations including mean and median lifespan (days), as well as XiSquare value and p-Value using log rank test. D. Western blot showing GFP levels in wild-type (no GFP) whole larvae as well as esgGal4, UAS-GFP whole larvae with (esgGal4, UAS-GFP; tubGal80^ts^) and without the Gal80 repressor. At a permissive temperature (22 ° C), no GFP is detected in the presence of ubGal80^ts^. E. Shifting adult flies (esgGal4, UAS-GFP; tubGal80^ts^) to 29 ° C strongly induces GFP expression in the gut.
(0.94 MB TIF)

Figure S3 ISC are the only proliferating cells in the midgut epithelium. A. Confocal image of a representative MARCM clone induced in the intestinal epithelium. The arrowhead indicates the unique pH3+ cell among GFP+ cells. B. Analysis of proliferating cells in the ISC lineage in young (7 days), old (40 days) or stressed flies, as well as ISC over-expressing Hep. For each condition, MARCM clones were observed 7 days after induction. The number of clones containing 1 pH3+ cell, 2 pH3+ cells or more is indicated. The size of the clones is also reported. No clones with more than 2 pH3+ cells could be detected in any of the conditions tested (>200 clones observed for each), and the clones induced in older or stressed animals are not larger than the clones induced in young animals, suggesting the absence of transient amplifying cells, even in older animals.
(1.00 MB TIF)

Figure S4 qRT-PCR confirming the reduction of Akt expression using the UAS-Akt^RNAi^ line. RNA was obtained from wild-type and daG4>UAS-Akt^RNAi^ embryos, at 25 ° C (Daughterless-Gal4 (daG4) is a ubiquitous driver). Actin5C served as internal control. Bars represent the average of 3 independent samples ± standard deviation, and p-value is calculated using Student's t-test.
(0.08 MB TIF)

Figure S5 Lifespan analysis of esgGal4 heterozygous flies. A. Survival curves of esgGal4/+ flies compared to their respective wild-type co-isogenic controls. The esgGal4 line was backcrossed 10 generation into *w* ^1118^ background. esgGal4/+ females were crossed to *y* ^1^ *w* ^1^ males and survival of the progeny was recorded. These flies represent the isogenic controls for the experiment presented in Figure 3D. B. Summary of the lifespan analysis of esgGal4/+ flies. See Table S3 for complete analysis.
(0.33 MB TIF)

Figure S6 Expression pattern of the 5961GeneSwitch driver. A. Western-blot analysis of dissected tissues from adult 5961GS>GFP females. GFP can exclusively be detected in the intestine after exposure to RU486. Heterochromatin Protein 1 (detected by immuno-staining) and total protein levels (Ponceau Red staining) are shown as loading controls. B. Bright field and fluorescence images of dissected esgGal4>GFP and 5961GS>GFP (fed RU486 for 3 days) females, confirming that 5961-driven transgene expression is much weaker than esg-mediated expression. C. Western-blot analysis of head extracts from adult 5961GS>GFP females, compared to standard neuronal drivers (elavGal4 and elavGeneSwitch), as well as esgGal4. No GFP can be detected in the extracts from 5961GS>GFP flies, further suggesting that the activity of the 5961GS driver is restricted to the intestine and the malpighian tubules.
(4.96 MB TIF)

Figure S7 Induction of Hsp68 and Jafrac1 by Foxo. qRT-PCR demonstrating induction of hsp68 and Jafrac1 in response to expression of constitutively active Foxo (Foxo^TM^) in third instar eye imaginal discs, using the sepGal4 driver. Average and standard-deviation from 3 independent experiments are shown. p-value is calculated using Student's t-test. RNA was collected from 10 discs for each experiment. Expression levels are reported as relative to actin5C expression.
(0.15 MB TIF)

Figure S8 Overexpression of stress-protective genes in ISC lineage delays dysplasia in the anterior midgut. A. The age-related loss of tissue can be scored in the anterior midgut. This phenotype can be scored using three distinct categories, based the presence of individual esg+ cells (category 1), the formation of esg+ cell clusters (category 2) or the invasion of the proventriculus by esg+ cells (category 3). The white bars mark the limit between the anterior midgut and the proventriculus (PV). B. Intestinal degeneration in aging (3, 35, and 50 days) control flies (esgGFP>+) and flies overexpressing cytoprotective genes in the ISCs (esgGFP>Jafrac1 and esgGFP>Hsp68) was scored using the method described above. Overexpression of Jafrac1 or Hsp68 delays age-related loss of intestinal architecture in the anterior midgut. p-value from Pearson XiSquare test.
(0.67 MB TIF)

Figure S9 Expression of Hsp68 under the control of the GMRGal4 driver doesn't affect lifespan. A. Survival curves of GMRGal4> Hsp68 compared to their respective wild type co-isogenic controls. The UAS-Hsp68 line was backcrossed 10 generation into *w* ^1118^ background. Wild type and UAS siblings were crossed to GMRGal4,UAS-GFP and survival of the progeny was recorded. B. Summary of the lifespan analysis of GMRGAl4> Hsp68. No significant difference in longevity was observed between Hsp68 over-expressing flies and their controls.
(0.26 MB TIF)

Table S1 Lifespan analysis of flies with impaired intestinal regeneration. Sex, genotypes, and mean lifespan statistics of the populations used for demographic analysis (Figure 1E) are listed. Experimental and control populations are compared using Log-Rank and Wilcoxon tests (ChiSquare and p-values). All the analysis was performed using the JMP7 statistical software.
(0.26 MB PDF)

Table S2 Lifespan analysis of flies with strong reduction of IIS using the esgGal4 driver. Sex, genotypes and mean lifespan statistics of the populations used for demographic analysis (Figure 3B and 3C) are listed. Experimental and control populations are compared using Log-Rank and Wilcoxon tests. All the analysis was performed using the JMP7 statistical software.
(0.25 MB PDF)

Table S3 Lifespan analysis of flies overexpressing Foxo using the esgGal4 driver and corresponding controls. Sex, genotypes, and lifespan statistics of individual cohorts used for demographic analysis (Figure 3D) are listed. Mean lifespan and days at which 25% or 75% of the population were dead are shown for each cohort. Flies from the same population are siblings from individual crosses. ChiSquare and p-values are derived from Log-Rank and Wilcoxon Tests. All the analysis was performed using the JMP7 statistical software.
(0.35 MB PDF)

Table S4 Lifespan analysis of flies with moderate reduction of IIS and JNK signaling using the 5961GS driver. Genotypes and lifespan statistics of individual cohorts used for demographic analysis in Figure 4 are listed. Mean lifespan, median lifespan and days at which 25% or 75% of the population were dead are shown for each cohort. Flies from the same population (-RU486 and +RU486) are siblings from individual crosses. Only females are shown. The significance of the changes observed when the flies are raised on RU486 was tested using Log-Rank and Wilcoxon Tests (ChiSquare and p-values). All the analysis was performed using the JMP7 statistical software.
(0.35 MB PDF)

Table S5 Lifespan analysis of flies with increased stress protection using the esgGal4 driver (esgGal4,GFP> Jafrac1 and esgGal4,GFP> Hsp68). Sex, genotypes, and lifespan statistics of individual cohorts used for demographic analysis (Figure 6A) are listed. Mean and median lifespan and days at which 25% or 75% of the population were dead are shown for each cohort. ChiSquare and p values are derived from Log-Rank and Wilcoxon Tests. All the analysis was performed using the JMP7 statistical software.
(0.34 MB PDF)

Table S6 Lifespan analysis of flies with moderate expression of Jafrac1 and Hsp68 using the 5961GS driver. Genotypes and lifespan statistics of individual cohorts used for demographic analysis (Figure 6C) are listed. Mean and median lifespan and days at which 25% or 75% of the population were dead are shown for each cohort. Flies from the same population (-RU486 and +RU486) are siblings from individual crosses. Only females are shown. The significance of the changes observed when the flies are raised on RU486 was tested using Log-Rank and Wilcoxon Tests (ChiSquare and p-values). All the analysis was performed using the JMP7 statistical software.
(0.34 MB PDF)

Text S1 Description of scoring methods used to monitor intestinal degeneration in aging flies.
(0.03 MB DOC)
