# Supplementary figures and images for: Lifespan Extension by Preserving Proliferative Homeostasis in Drosophila
Source: PLoS Genet. 2010 Oct 14;6(10):e1001159. doi: 10.1371/journal.pgen.1001159 (PMC2954830; doi:10.1371/journal.pgen.1001159)

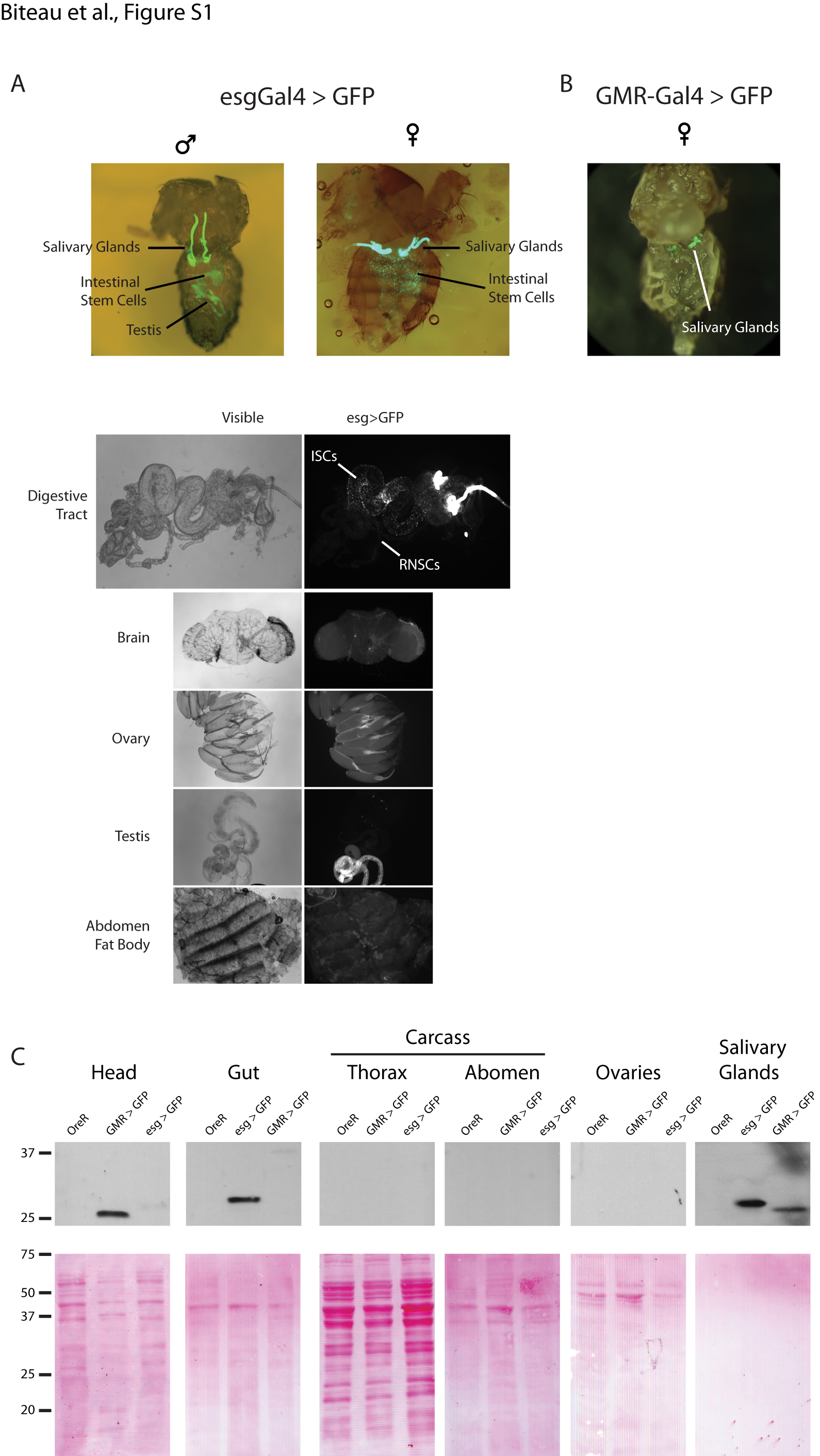

Supplement: Figure S1 — Expression pattern of the esgGal4 driver. A. All accessible tissues from flies expressing GFP under the control of the esgGal4 driver were dissected. GFP can exclusively be detected in the Intestinal Stem Cells (ISCs) in the midgut, the Renal Stem Cells (RNSCs) in the malpighian tubules, the testis and the salivary glands. No Fluorescence can be detected in the crop, the hindgut, the ovaries, the brain or the abdominal fat body. B. GMRGal4, used as a control for lifespan experiments, overlaps with esgGal4 expression in the salivary glands. C. Western-blot analysis of dissected tissues from adults in which esgGal4 or GMRGal4 drive expression of GFP. Extracts from OreR (wild-type) flies are shown as controls. Note the overlap of expression of the two drivers in salivary glands, and the exclusive expression of esg-GFP in the intestine and salivary glands. Mobility shift between the GFPs expressed in either line is due to expression of different GFP constructs in esgGal4 or GMRGal4 recombinants. Ponceau Red staining is shown as loading control. (4.01 MB TIF) [file pgen.1001159.s001.tif]

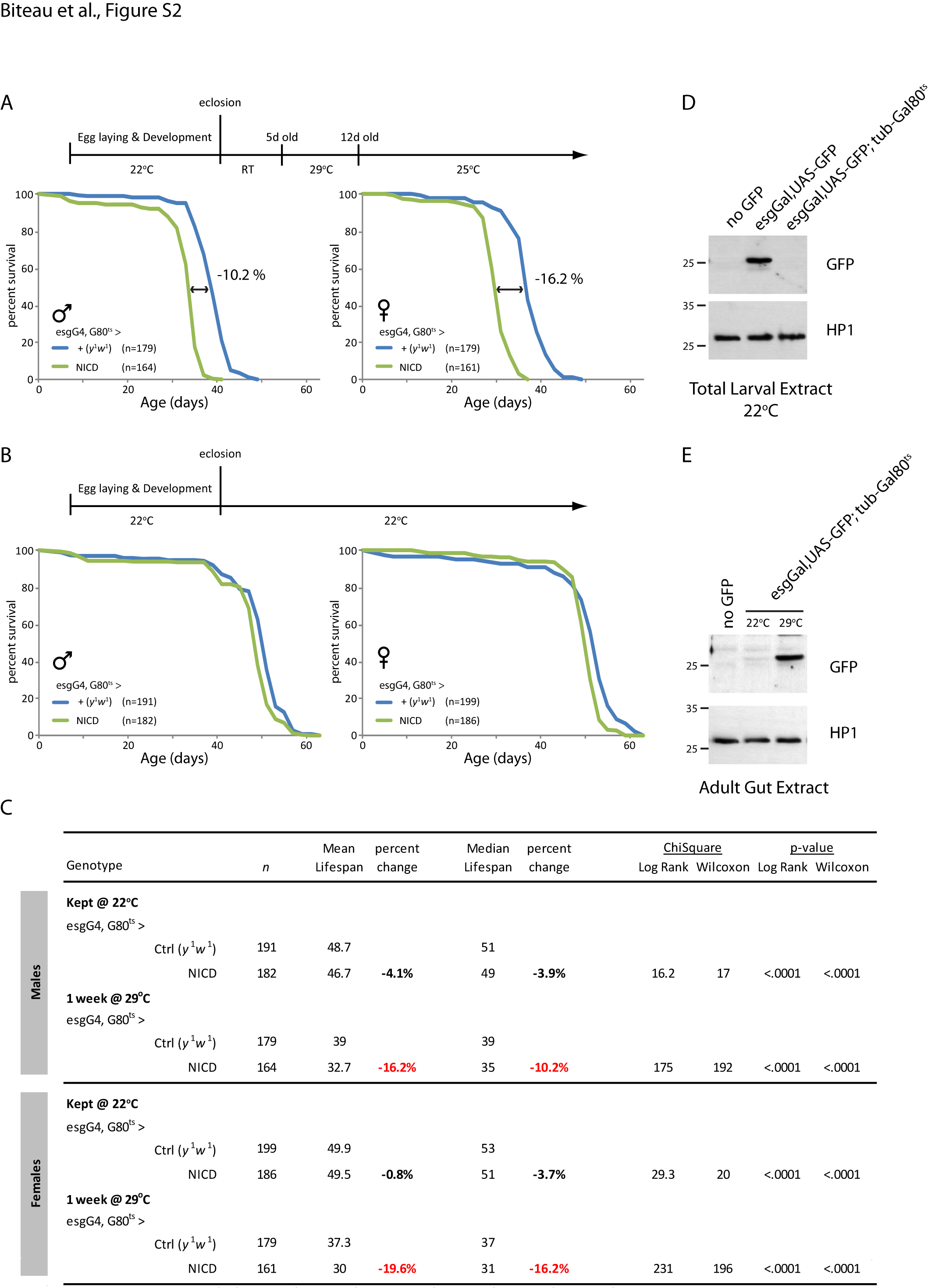

Supplement: Figure S2 — ISC function is critical for normal lifespan. A. Inducing differentiation in the ISC lineage (using activated notch intra-cellular domain; esgGal4, UAS-GFP;tubGal80ts/UAS-NICD) results in shortening of lifespan compared to wild-type controls (esgGal4, UAS-GFP;tubGal80ts/+ (y 1 w 1)) in both males and females. Flies used in these experiments were reared at a permissive temperature (22 ° C, promoting activity of the Gal80 repressor), then transiently shifted to 29 ° C at 5 days of age (for 7 days) to deactivate the repressor and irreversibly impair stem cell function through over-expression of NICD. Flies were aged at 25 ° C. B. Longevity (of esgGal4, UAS-GFP; tubGal80ts/UAS-NICD) is minimally affected when flies are kept at a permissive temperature (22 ° C, promoting activity of the Gal80 repressor) throughout life. This suggests that the TARGET system (tubGal80ts) approach can inhibit developmental effects of transgenes, thus limiting the lifespan effects strictly to changes in the adult. C. Summary of lifespan statistics for all populations including mean and median lifespan (days), as well as XiSquare value and p-Value using log rank test. D. Western blot showing GFP levels in wild-type (no GFP) whole larvae as well as esgGal4, UAS-GFP whole larvae with (esgGal4, UAS-GFP; tubGal80ts) and without the Gal80 repressor. At a permissive temperature (22 ° C), no GFP is detected in the presence of ubGal80ts. E. Shifting adult flies (esgGal4, UAS-GFP; tubGal80ts) to 29 ° C strongly induces GFP expression in the gut. (0.94 MB TIF) [file pgen.1001159.s002.tif]

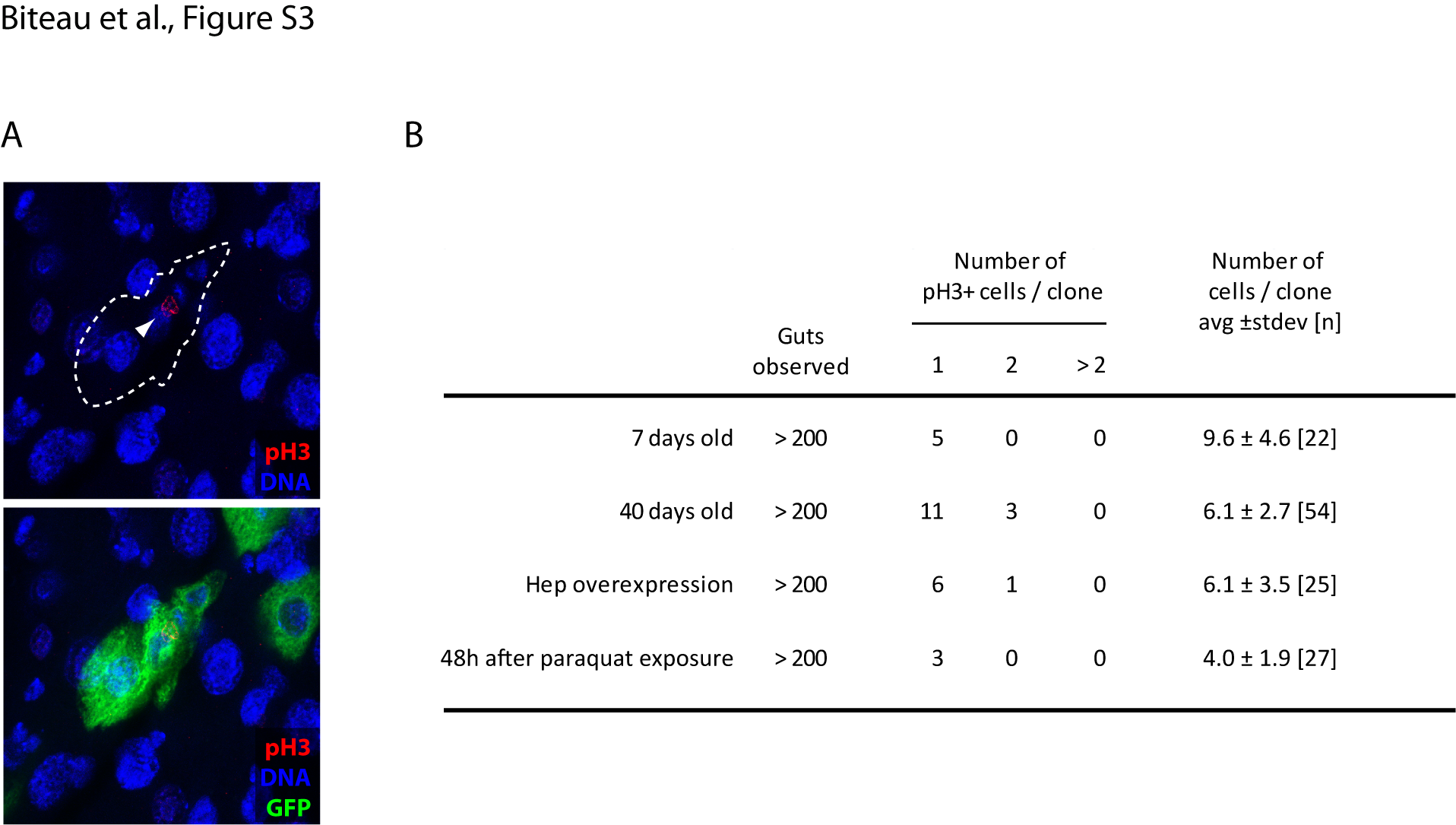

Supplement: Figure S3 — ISC are the only proliferating cells in the midgut epithelium. A. Confocal image of a representative MARCM clone induced in the intestinal epithelium. The arrowhead indicates the unique pH3+ cell among GFP+ cells. B. Analysis of proliferating cells in the ISC lineage in young (7 days), old (40 days) or stressed flies, as well as ISC over-expressing Hep. For each condition, MARCM clones were observed 7 days after induction. The number of clones containing 1 pH3+ cell, 2 pH3+ cells or more is indicated. The size of the clones is also reported. No clones with more than 2 pH3+ cells could be detected in any of the conditions tested (>200 clones observed for each), and the clones induced in older or stressed animals are not larger than the clones induced in young animals, suggesting the absence of transient amplifying cells, even in older animals. (1.00 MB TIF) [file pgen.1001159.s003.tif]

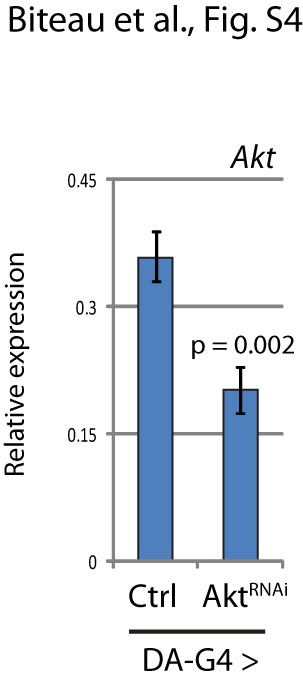

Supplement: Figure S4 — qRT-PCR confirming the reduction of Akt expression using the UAS-AktRNAi line. RNA was obtained from wild-type and daG4>UAS-AktRNAi embryos, at 25 ° C (Daughterless-Gal4 (daG4) is a ubiquitous driver). Actin5C served as internal control. Bars represent the average of 3 independent samples ± standard deviation, and p-value is calculated using Student's t-test. (0.08 MB TIF) [file pgen.1001159.s004.tif]

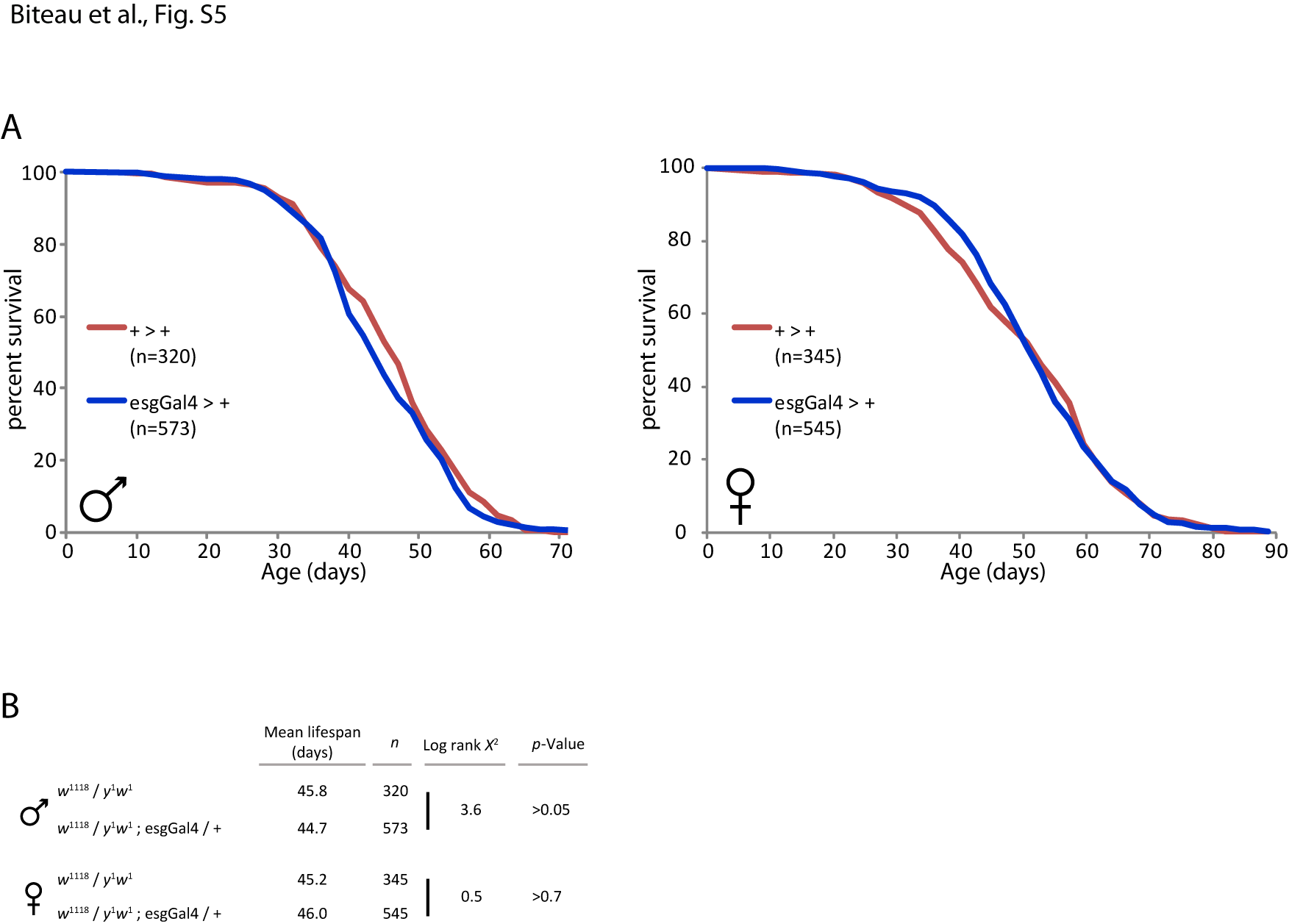

Supplement: Figure S5 — Lifespan analysis of esgGal4 heterozygous flies. A. Survival curves of esgGal4/+ flies compared to their respective wild-type co-isogenic controls. The esgGal4 line was backcrossed 10 generation into w 1118 background. esgGal4/+ females were crossed to y 1 w 1 males and survival of the progeny was recorded. These flies represent the isogenic controls for the experiment presented in Figure 3D. B. Summary of the lifespan analysis of esgGal4/+ flies. See Table S3 for complete analysis. (0.33 MB TIF) [file pgen.1001159.s005.tif]

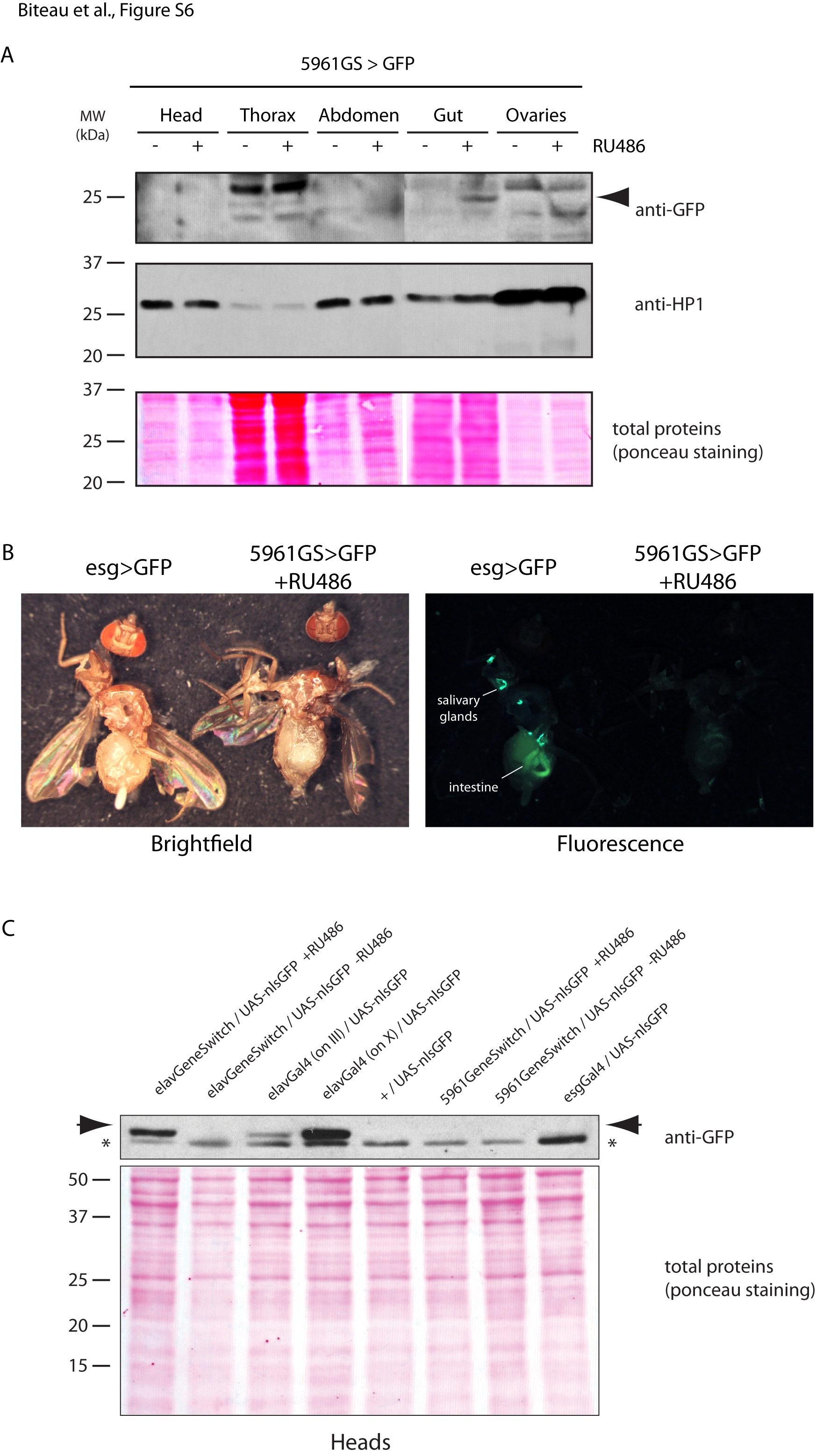

Supplement: Figure S6 — Expression pattern of the 5961GeneSwitch driver. A. Western-blot analysis of dissected tissues from adult 5961GS>GFP females. GFP can exclusively be detected in the intestine after exposure to RU486. Heterochromatin Protein 1 (detected by immuno-staining) and total protein levels (Ponceau Red staining) are shown as loading controls. B. Bright field and fluorescence images of dissected esgGal4>GFP and 5961GS>GFP (fed RU486 for 3 days) females, confirming that 5961-driven transgene expression is much weaker than esg-mediated expression. C. Western-blot analysis of head extracts from adult 5961GS>GFP females, compared to standard neuronal drivers (elavGal4 and elavGeneSwitch), as well as esgGal4. No GFP can be detected in the extracts from 5961GS>GFP flies, further suggesting that the activity of the 5961GS driver is restricted to the intestine and the malpighian tubules. (4.96 MB TIF) [file pgen.1001159.s006.tif]

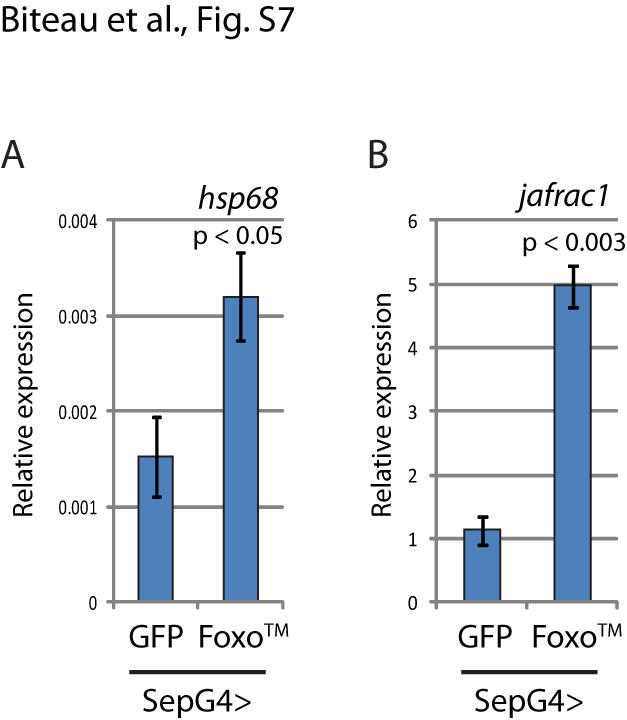

Supplement: Figure S7 — Induction of Hsp68 and Jafrac1 by Foxo. qRT-PCR demonstrating induction of hsp68 and Jafrac1 in response to expression of constitutively active Foxo (FoxoTM) in third instar eye imaginal discs, using the sepGal4 driver. Average and standard-deviation from 3 independent experiments are shown. p-value is calculated using Student's t-test. RNA was collected from 10 discs for each experiment. Expression levels are reported as relative to actin5C expression. (0.15 MB TIF) [file pgen.1001159.s007.tif]

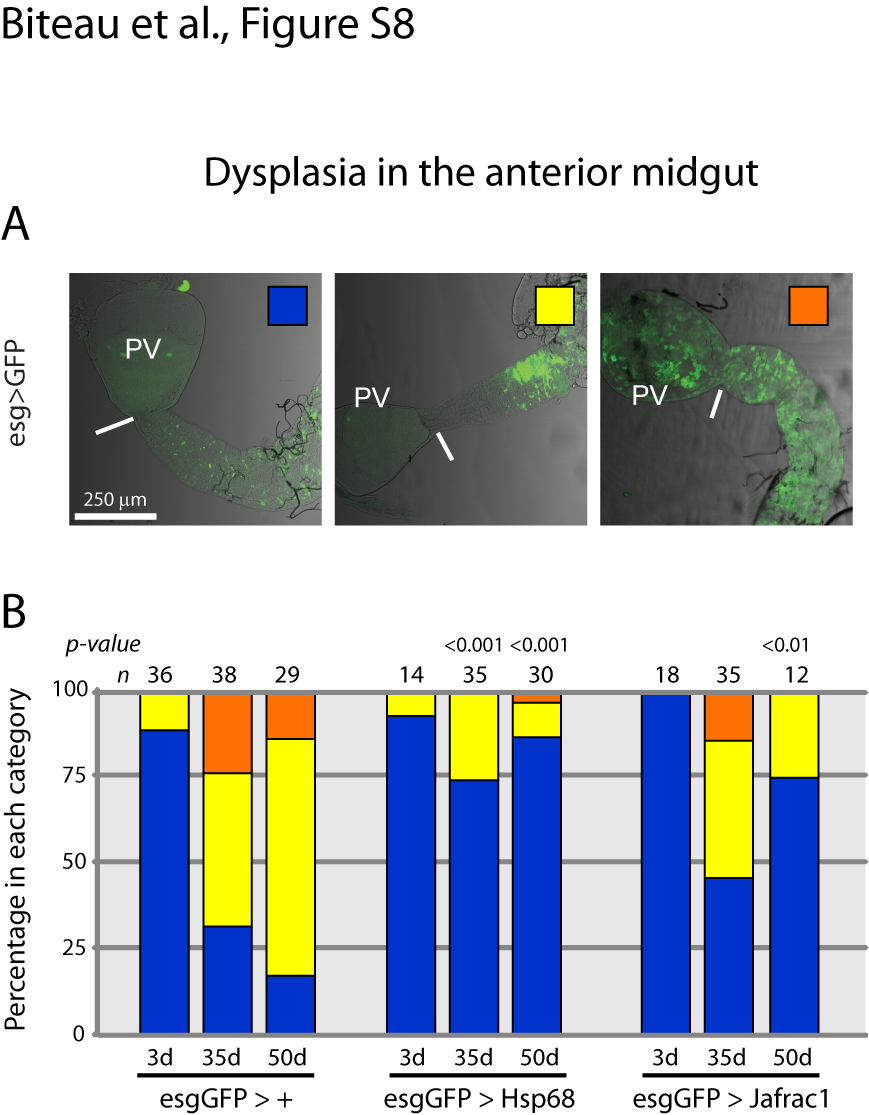

Supplement: Figure S8 — Overexpression of stress-protective genes in ISC lineage delays dysplasia in the anterior midgut. A. The age-related loss of tissue can be scored in the anterior midgut. This phenotype can be scored using three distinct categories, based the presence of individual esg+ cells (category 1), the formation of esg+ cell clusters (category 2) or the invasion of the proventriculus by esg+ cells (category 3). The white bars mark the limit between the anterior midgut and the proventriculus (PV). B. Intestinal degeneration in aging (3, 35, and 50 days) control flies (esgGFP>+) and flies overexpressing cytoprotective genes in the ISCs (esgGFP>Jafrac1 and esgGFP>Hsp68) was scored using the method described above. Overexpression of Jafrac1 or Hsp68 delays age-related loss of intestinal architecture in the anterior midgut. p-value from Pearson XiSquare test. (0.67 MB TIF) [file pgen.1001159.s008.tif]

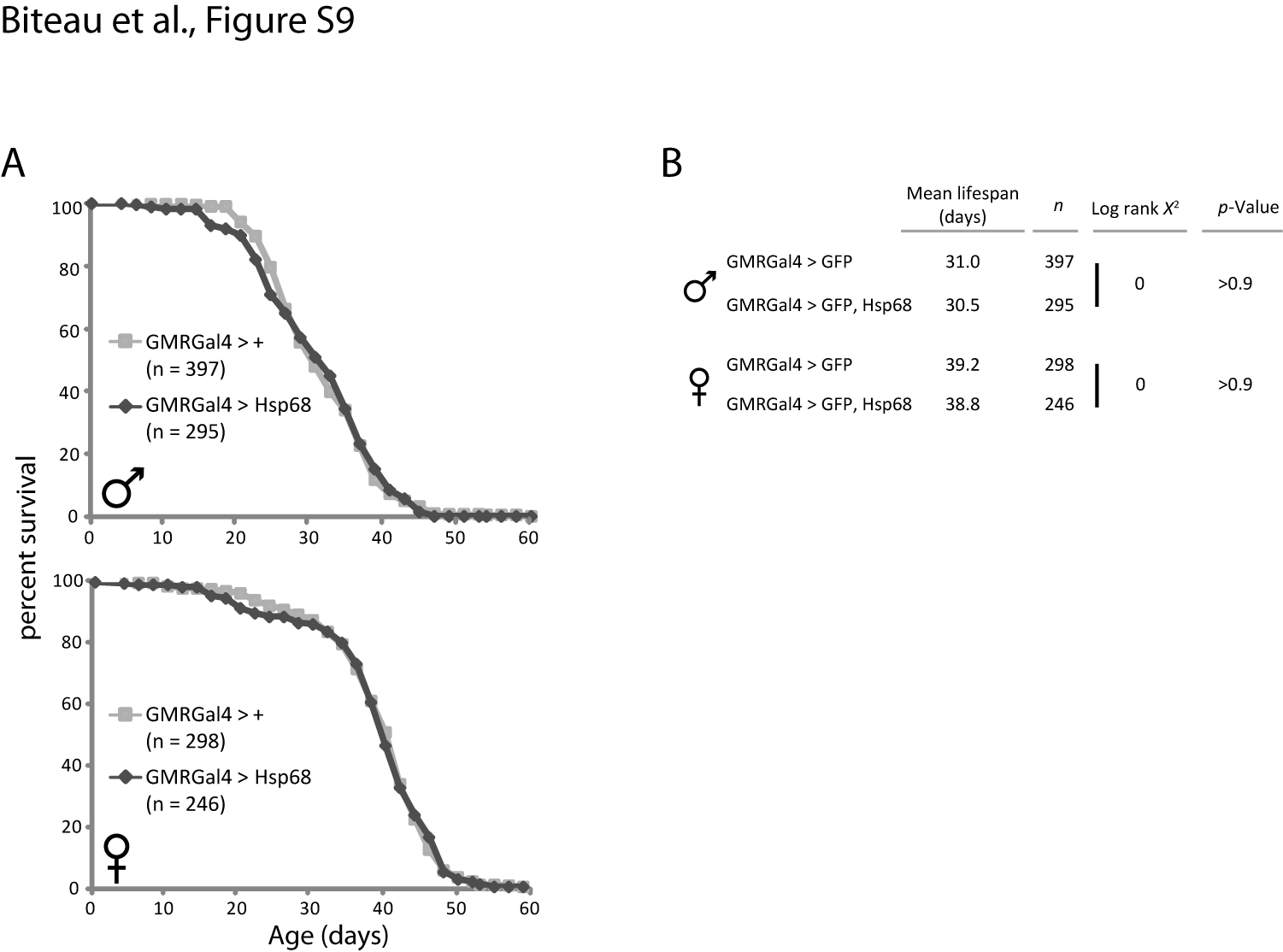

Supplement: Figure S9 — Expression of Hsp68 under the control of the GMRGal4 driver doesn't affect lifespan. A. Survival curves of GMRGal4> Hsp68 compared to their respective wild type co-isogenic controls. The UAS-Hsp68 line was backcrossed 10 generation into w 1118 background. Wild type and UAS siblings were crossed to GMRGal4,UAS-GFP and survival of the progeny was recorded. B. Summary of the lifespan analysis of GMRGAl4> Hsp68. No significant difference in longevity was observed between Hsp68 over-expressing flies and their controls. (0.26 MB TIF) [file pgen.1001159.s009.tif]
