# Supplementary material for: Lifespan Extension by Preserving Proliferative Homeostasis in Drosophila
Source: PLoS Genet. 2010 Oct 14;6(10):e1001159. doi: 10.1371/journal.pgen.1001159 (PMC2954830; doi:10.1371/journal.pgen.1001159)
Supplement: Table S1 — Lifespan analysis of flies with impaired intestinal regeneration. Sex, genotypes, and mean lifespan statistics of the populations used for demographic analysis (Figure 1E) are listed. Experimental and control populations are compared using Log-Rank and Wilcoxon tests (ChiSquare and p-values). All the analysis was performed using the JMP7 statistical software. (0.26 MB PDF) [file pgen.1001159.s010.pdf]

	Genotype	n	Mean Lifespan	percent extension	ChiSquare Log Rank	ChiSquare Wilcoxon	p-value Log Rank	p-value Wilcoxon
**Males**	esgG4, G80^{ts} >							
	Ctrl (w^{1118})	493	26.9					
	Hep^{WT}	445	25.2	**-6.3%**	153	140	<.0001	<.0001
	InR^{WT}	321	25.2	**-6.3%**	138	127	<.0001	<.0001
	Bsk^{RNAi}	439	22.5	**-16.4%**	657	594	<.0001	<.0001
**Females**	esgG4, G80^{ts} >							
	Ctrl (w^{1118})	473	26.4					
	Hep^{WT}	376	24.9	**-5.7%**	83	79	<.0001	<.0001
	InR^{WT}	310	23.7	**-10.2%**	199	164	<.0001	<.0001
	Bsk^{RNAi}	301	23.7	**-10.2%**	307	265	<.0001	<.0001
