# Supplementary material for: Lifespan Extension by Preserving Proliferative Homeostasis in Drosophila
Source: PLoS Genet. 2010 Oct 14;6(10):e1001159. doi: 10.1371/journal.pgen.1001159 (PMC2954830; doi:10.1371/journal.pgen.1001159)
Supplement: Table S2 — Lifespan analysis of flies with strong reduction of IIS using the esgGal4 driver. Sex, genotypes and mean lifespan statistics of the populations used for demographic analysis (Figure 3B and 3C) are listed. Experimental and control populations are compared using Log-Rank and Wilcoxon tests. All the analysis was performed using the JMP7 statistical software. (0.25 MB PDF) [file pgen.1001159.s011.pdf]

	Genotype	*n*	Mean Lifespan	percent extension	ChiSquare Log Rank	ChiSquare Wilcoxon	p-value Log Rank	p-value Wilcoxon
**Males**	esgG4, $G80^{ts}$ >							
	Ctrl (w^{1118})	333	27.2					
	Akt^{RNAi}	339	24.1	**-11.4%**	325	283	<.0001	<.0001
	Ctrl ($y^1 w^1$)	357	28.2					
	Foxo	394	26.4	**-6.4%**	160	145	<.0001	<.0001
	$Dp110^{DN}$	380	27.4	**-2.8%**	47	42	<.0001	<.0001
**Females**	esgG4, $G80^{ts}$ >							
	Ctrl (w^{1118})	263	27.2					
	Akt^{RNAi}	351	25.2	**-7.4%**	161	136	<.0001	<.0001
	Ctrl ($y^1 w^1$)	373	27.8					
	Foxo	373	26.6	**-4%**	75	74	<.0001	<.0001
	$Dp110^{DN}$	337	26.5	**-5%**	69	61	<.0001	<.0001
