# Supplementary material for: Lifespan Extension by Preserving Proliferative Homeostasis in Drosophila
Source: PLoS Genet. 2010 Oct 14;6(10):e1001159. doi: 10.1371/journal.pgen.1001159 (PMC2954830; doi:10.1371/journal.pgen.1001159)
Supplement: Table S3 — Lifespan analysis of flies overexpressing Foxo using the esgGal4 driver and corresponding controls. Sex, genotypes, and lifespan statistics of individual cohorts used for demographic analysis (Figure 3D) are listed. Mean lifespan and days at which 25% or 75% of the population were dead are shown for each cohort. Flies from the same population are siblings from individual crosses. ChiSquare and p-values are derived from Log-Rank and Wilcoxon Tests. All the analysis was performed using the JMP7 statistical software. (0.35 MB PDF) [file pgen.1001159.s012.pdf]

esgGal4 > + (y^1w^1/w^{1118} outcrossed background)

Population	Sex	Genotype		*n*	Mean Lifespan	25% dead	75% dead	ChiSquare Log Rank	ChiSquare Wilcoxon	p-value Log Rank	p-value Wilcoxon
A	Males	+ > +		35	47.3	40	57	0.7	0.1	0.38	0.73
		esgGal4 > +		83	47.3	42	55				
			percent extension		***0.0%***	***5.0%***	***-3.5%***				
	Females	+ > +		54	53.3	47	61	1.2	1.8	0.28	0.17
		esgGal4 > +		101	51.2	45	59				
			percent extension		***-3.9%***	***-4.3%***	***-3.3%***				
B	Males	+ > +		64	45.1	38	51	6.4	8.4	0.01	0.004
		esgGal4 > +		94	50.2	42	55				
			percent extension		***11.3%***	***10.5%***	***7.8%***				
	Females	+ > +		46	46.3	34	53	0.1	0.2	0.72	0.66
		esgGal4 > +		93	45.8	40	55				
			percent extension		***-1.1%***	***17.6%***	***3.8%***				
C	Males	+ > +		46	47.2	42	53	0.7	4.6	0.03	0.03
		esgGal4 > +		78	44	38	49				
			percent extension		***-6.8%***	***-9.5%***	***-7.5%***				
	Females	+ > +		50	48.5	45	53	0.2	0.2	0.62	0.62
		esgGal4 > +		69	47.8	42	55				
			percent extension		***-1.4%***	***-6.7%***	***3.8%***				
D	Males	+ > +		64	45.4	38	53	21.4	12.3	<0.0001	0.0004
		esgGal4 > +		107	39.7	38	45				
			percent extension		***-12.6%***	***0.0%***	***-15.1%***				
	Females	+ > +		70	42.7	36	53	2	6.2	0.15	0.01
		esgGal4 > +		99	46.4	42	51				
			percent extension		***8.7%***	***16.7%***	***-3.8%***				
E	Males	+ > +		62	45.7	40	53	12	24.6	0.005	<0.0001
		esgGal4 > +		93	38.2	30	45				
			percent extension		***-16.4%***	***-25.0%***	***-15.1%***				
	Females	+ > +		68	45.6	38	53	0.7	2	0.38	0.16
		esgGal4 > +		78	43.5	38	51				
			percent extension		***-4.6%***	***0.0%***	***-3.8%***				
F	Males	+ > +		49	44.7	38	51	1	5	0.3	0.02
		esgGal4 > +		118	48.4	45	55				
			percent extension		***8.3%***	***18.4%***	***7.8%***				
	Females	+ > +		57	36.2	28	40	5.7	16.3	0.02	<0.0001
		esgGal4 > +		105	41.7	36	47				
			percent extension		***15.2%***	***28.6%***	***17.5%***				
**Total**	**Males**	**+ > +**		**320**	**45.8**	**38**	**53**	**3.6**	**3.7**	**0.06**	**0.05**
		**esgGal4 > +**		**573**	**44.7**	**38**	**53**				
			percent extension		***-2.4%***	***0.0%***	***0.0%***				
	**Females**	**+ > +**		**345**	**45.2**	**36**	**53**	**0.1**	**0.5**	**0.79**	**0.44**
		**esgGal4 > +**		**545**	**46**	**40**	**53**				
			percent extension		***1.8%***	***11.1%***	***0.0%***				

esgGal4 > Foxo ($y^1 w^1$ / w^{1118} outcrossed background)

Population	Sex	Genotype	*n*	Mean Lifespan	25% dead	75% dead	ChiSquare Log Rank	ChiSquare Wilcoxon	p-value Log Rank	p-value Wilcoxon
A	Males	+ > Foxo	30	53.9	51	61	41.7	36.2	<0.0001	<0.0001
		esgGal4 > Foxo	34	35.6	29	47				
		percent extension		***-33.9%***	***-43.1%***	***-22.9%***				
	Females	+ > Foxo	41	58.7	53	67	9.5	13.3	0.002	0.0003
		esgGal4 > Foxo	43	47.1	37	61				
		percent extension		***-19.8%***	***-30.2%***	***-8.9%***				
B	Males	+ > Foxo	35	53.5	55	59	49.6	36.3	<0.0001	<0.0001
		esgGal4 > Foxo	46	38.7	35	47				
		percent extension		***-27.7%***	***-36.3%***	***-20.3%***				
	Females	+ > Foxo	22	60.7	53	75	0.2	0.1	0.64	0.82
		esgGal4 > Foxo	28	60.3	55	71				
		percent extension		***-0.6%***	***+3.7%***	***-5.3%***				
C	Males	+ > Foxo	42	48.6	43	55	18.9	19.6	<0.0001	<0.0001
		esgGal4 > Foxo	54	40.7	37	47				
		percent extension		***-16.3%***	***-13.9%***	***-14.5%***				
	Females	+ > Foxo	41	52.9	47	65	2.2	3.8	0.14	0.05
		esgGal4 > Foxo	46	48.9	39	57				
		percent extension		***-7.6%***	***-17%***	***-12.3%***				
D	Males	+ > Foxo	43	49.4	47	59	60.9	46	<0.0001	<0.0001
		esgGal4 > Foxo	43	34.5	29	41				
		percent extension		***-30.2%***	***-38.3%***	***-30.5%***				
	Females	+ > Foxo	36	55.7	55	63	16	18	<0.0001	<0.0001
		esgGal4 > Foxo	41	45	45	53				
		percent extension		***-19.2%***	***-18.2%***	***-15.9%***				
E	Males	+ > Foxo	39	49.8	49	59	41	31.3	<0.0001	<0.0001
		esgGal4 > Foxo	40	37.2	35	43				
		percent extension		***-25.3%***	***-28.6%***	***-27.1%***				
	Females	+ > Foxo	40	53.5	53	63	9.1	9.9	0.002	0.001
		esgGal4 > Foxo	37	48.1	45	53				
		percent extension		***-10.1%***	***-15.1%***	***-15.9%***				
**Total**	**Males**	**+ > Foxo**	**189**	**50.8**	**47**	**59**	**191.4**	**156.7**	**<0.0001**	**<0.0001**
		**esgGal4 > Foxo**	**217**	**37.6**	**33**	**45**				
		percent extension		***-26.0%***	***-29.8%***	***-23.7%***				
	**Females**	**+ > Foxo**	**179**	**55.7**	**53**	**65**	**24.2**	**32**	**<0.0001**	**<0.0001**
		**esgGal4 > Foxo**	**192**	**48.6**	**43**	**57**				
		percent extension		***-12.7%***	***-18.9%***	***-12.3%***				
