# Supplementary material for: Lifespan Extension by Preserving Proliferative Homeostasis in Drosophila
Source: PLoS Genet. 2010 Oct 14;6(10):e1001159. doi: 10.1371/journal.pgen.1001159 (PMC2954830; doi:10.1371/journal.pgen.1001159)
Supplement: Table S4 — Lifespan analysis of flies with moderate reduction of IIS and JNK signaling using the 5961GS driver. Genotypes and lifespan statistics of individual cohorts used for demographic analysis in Figure 4 are listed. Mean lifespan, median lifespan and days at which 25% or 75% of the population were dead are shown for each cohort. Flies from the same population (-RU486 and +RU486) are siblings from individual crosses. Only females are shown. The significance of the changes observed when the flies are raised on RU486 was tested using Log-Rank and Wilcoxon Tests (ChiSquare and p-values). All the analysis was performed using the JMP7 statistical software. (0.35 MB PDF) [file pgen.1001159.s013.pdf]

Genotype	Population	Drug	*n*	Mean Lifespan	25% dead	Median Lifespan	75% dead	ChiSquare Log Rank	ChiSquare Wilcoxon	p-value Log Rank	p-value Wilcoxon
5961 GeneSwitch > + (w^{1118})	A	-RU486	71	60.6	56	66	73	7.7	3.5	0.006	0.06
		+RU486	73	64.6	59	68	73				
	percent extension			***6.6%***	***5.4%***	***3.0%***	***0.0%***				
	B	-RU486	93	56.9	49	59	68	5.7	5.9	0.01	0.01
		+RU486	87	61	54	66	73				
	percent extension			***7.2%***	***10.2%***	***11.9%***	***7.4%***				
	C	-RU486	80	66.9	61	73	79	2.6	0.7	0.1	0.4
		+RU486	79	69.1	61	73	79				
	percent extension			***3.3%***	***0.0%***	***0.0%***	***0.0%***				
	D	-RU486	76	68.9	61	76	79	0.3	0	0.55	0.96
		+RU486	70	69.3	64	73	79				
	percent extension			***0.6%***	***4.9%***	***-3.9%***	***0.0%***				
	**Total**	**-RU486**	**320**	**63.1**	**54**	**67**	**75**	**7.5**	**4.5**	**0.006**	**0.03**
		**+RU486**	**309**	**65.8**	**59**	**68**	**76**				
	percent extension			***4.3%***	***9.3%***	***1.5%***	***1.3%***				
5961 GeneSwitch > + (OreR)	A	-RU486	133	65.8	61	68	71	15.3	20.9	<0.0001	<0.0001
		+RU486	132	68.9	68	71	71				
	percent extension			***4.7%***	11.5%	***4.4%***	***0.0%***				
	B	-RU486	117	65.9	61	68	71	4.8	4.7	0.03	0.03
		+RU486	126	66.3	64	68	74				
	percent extension			***0.6%***	***4.9%***	***0.0%***	***4.2%***				
	C	-RU486	166	65.4	62	66	72	15.6	17.4	<0.0001	<0.0001
		+RU486	154	68.5	66	72	72				
	percent extension			***4.7%***	***6.5%***	***9.1%***	***0.0%***				
	**Total**	**-RU486**	**416**	**65.7**	**62**	**68**	**71**	**30.9**	**39.6**	<0.0001	<0.0001
		**+RU486**	**412**	**67.9**	**66**	**69**	**72**				
	percent extension			***3.3%***	***6.5%***	***1.5%***	***1.4%***				

Genotype	Population	Drug	*n*	Mean Lifespan	25% dead	Median Lifespan	75% dead	ChiSquare Log Rank	ChiSquare Wilcoxon	p-value Log Rank	p-value Wilcoxon
5961 GeneSwitch > Dp110^{DN}	A	-RU486	75	75.7	75	75	80	17	10.8	<0.0001	<0.0001
		+RU486	72	78.1	75	80	84				
		percent extension		***3.2%***	***0.0%***	***6.7%***	***5.0%***				
	B	-RU486	79	81.4	79	83	85	66.4	60	<0.0001	<0.0001
		+RU486	75	89.1	87	92	94				
		percent extension		***9.5%***	***10.1%***	***10.8%***	***10.6%***				
	C	-RU486	79	80.8	79	81	83	85.4	70.2	**<0.0001**	**<0.0001**
		+RU486	72	88.2	87	92	92				
		percent extension		***9.2%***	***10.1%***	***13.6%***	***10.8%***				
	**Total**	**-RU486**	**233**	**79.4**	**76**	**80**	**83**	**126.2**	**95.4**	**<0.0001**	**<0.0001**
		**+RU486**	**219**	**85.2**	**81**	**89**	**92**				
		percent extension		***7.3%***	***6.6%***	***11.3%***	***10.8%***				
5961 GeneSwitch > InR^{DN}	A	-RU486	32	69.5	75	80	80	11.2	17.6	0.0008	<0.0001
		+RU486	38	80.4	82	84	89				
		percent extension		***15.7%***	***9.3%***	***5.0%***	***11.3%***				
	B	-RU486	67	79.5	79	79	90	27.8	32.1	<0.0001	<0.0001
		+RU486	79	86.7	85	90	92				
		percent extension		***9.1%***	***7.6%***	***13.9%***	***2.2%***				
	C	-RU486	78	83	79	83	87	20.7	16	<0.0001	<0.0001
		+RU486	87	86.6	79	90	94				
		percent extension		***4.3%***	***0.0%***	***8.4%***	***8.0%***				
	D	-RU486	90	83.5	79	83	90	27.7	32.1	<0.0001	<0.0001
		+RU486	88	88.5	87	90	92				
		percent extension		***6.0%***	***10.1%***	***8.4%***	***2.2%***				
	**Total**	**-RU486**	**267**	**80.6**	**79**	**81**	**87**	**80.9**	**85**	**<0.0001**	**<0.0001**
		**+RU486**	**292**	**86.4**	**83**	**90**	**92**				
		percent extension		***7.2%***	***5.1%***	***11.1%***	***5.7%***				
5961 GeneSwitch > Akt^{RNAi}	A	-RU486	75	66.7	66	70	75	80.6	63.6	<0.0001	<0.0001
		+RU486	72	77.5	77	82	87				
		percent extension		***16.2%***	***16.7%***	***17.1%***	***16.0%***				
	B	-RU486	84	68.7	66	73	75	11.9	2.9	0.0006	0.086
		+RU486	83	69.5	63	75	77				
		percent extension		***1.2%***	***-4.5%***	***2.7%***	***2.7%***				
	C	-RU486	114	70	67	70	73	105.3	98.5	<0.0001	<0.0001
		+RU486	106	79	76	81	83				
		percent extension		***12.9%***	***13.4%***	***15.7%***	***13.7%***				
	D	-RU486	115	62.5	58	64	70	51.6	54.4	<0.0001	<0.0001
		+RU486	109	71.1	67	73	76				
		percent extension		***13.8%***	***15.5%***	***14.1%***	***8.6%***				
	E	-RU486	72	69.4	67	73	76	32.4	26.7	<0.0001	<0.0001
		+RU486	65	74.3	73	79	81				
		percent extension		***7.1%***	***9.0%***	***8.2%***	***6.6%***				
	**Total**	**-RU486**	**453**	**67.2**	**66**	**70**	**73**	**244.4**	**180.6**	**<0.0001**	**<0.0001**
		**+RU486**	**442**	**74.3**	**73**	**77**	**81**				
		percent extension		***10.6%***	***10.6%***	***10.0%***	***11.0%***				

Population	Drug	*n*	Mean Lifespan	25% dead	Median Lifespan	75% dead	ChiSquare Log Rank	ChiSquare Wilcoxon	p-value Log Rank	p-value Wilcoxon
**5961 GeneSwitch >Bsk^{DN}**										
A	-RU486	73	60.7	55	64	73	17.1	12.7	**<0.0001**	**0.0004**
	+RU486	70	68.2	64	73	78				
	percent extension		***12.4%***	***16.4%***	***14.1%***	***6.8%***				
B	-RU486	79	66.5	64	67	73	33.7	36.3	**<0.0001**	**0.0004**
	+RU486	79	76.3	73	76	87				
	percent extension		***14.7%***	***14.1%***	***13.4%***	***19.2%***				
C	-RU486	104	45.7	42	44	49	67.8	47.7	**<0.0001**	**0.0004**
	+RU486	105	55.1	47	54	63				
	percent extension		***20.6%***	***11.9%***	***22.7%***	***28.6%***				
D	-RU486	85	42.3	37	42	47	17.5	10.5	**<0.0001**	**0.0001**
	+RU486	82	47.4	40	47	56				
	percent extension		***12.1%***	***8.1%***	***11.9%***	***19.1%***				
**Total**	**-RU486**	**341**	**54.1**	**44**	**51**	**64**	**54.3**	**46.5**	<0.0001	<0.0001
	**+RU486**	**340**	**61.6**	**51**	**63**	**75**				
	percent extension		*13.9%*	*15.9%*	*23.5%*	*17.2%*				
**5961 GeneSwitch >Bsk^{RNAi}**										
A	-RU486	77	63.6	64	70	73	9.5	7	**0.002**	**0.008**
	+RU486	81	66.8	64	70	76				
	percent extension		***5.0%***	***0.0%***	***0.0%***	***4.1%***				
B	-RU486	128	51.7	44	54	56	52.3	24.3	**<0.0001**	**<0.0001**
	+RU486	107	54.2	49	58	63				
	percent extension		***4.8%***	***11.4%***	***7.4%***	***12.5%***				
C	-RU486	105	47.5	42	49	54	90	69.1	**<0.0001**	**<0.0001**
	+RU486	110	57.5	54	58	65				
	percent extension		***21.1%***	***28.6%***	***18.4%***	***20.4%***				
D	-RU486	112	36.2	30	37	42	42.3	31.8	**<0.0001**	**<0.0001**
	+RU486	113	42.6	37	42	49				
	percent extension		***17.7%***	***23.3%***	***13.5%***	***16.7%***				
**Total**	**-RU486**	**422**	**48.7**	**40**	**49**	**56**	**41.3**	**38.5**	<0.0001	<0.0001
	**+RU486**	**411**	**54.4**	**42**	**56**	**65**				
	percent extension		*11.7%*	*5.0%*	*14.3%*	*16.1%*				
