# Supplementary material for: Lifespan Extension by Preserving Proliferative Homeostasis in Drosophila
Source: PLoS Genet. 2010 Oct 14;6(10):e1001159. doi: 10.1371/journal.pgen.1001159 (PMC2954830; doi:10.1371/journal.pgen.1001159)
Supplement: Table S5 — Lifespan analysis of flies with increased stress protection using the esgGal4 driver (esgGal4,GFP> Jafrac1 and esgGal4,GFP> Hsp68). Sex, genotypes, and lifespan statistics of individual cohorts used for demographic analysis (Figure 6A) are listed. Mean and median lifespan and days at which 25% or 75% of the population were dead are shown for each cohort. ChiSquare and p values are derived from Log-Rank and Wilcoxon Tests. All the analysis was performed using the JMP7 statistical software. (0.34 MB PDF) [file pgen.1001159.s014.pdf]

	Population	Sex	Genotype	*n*	Mean Lifespan	25% dead	Median Lifespan	75% dead	ChiSquare Log Rank	ChiSquare Wilcoxon	p-value Log Rank	p-value Wilcoxon
**esgGFP > Jafrac1**	A	Males	esgGal4,UAS-GFP > +	217	48.5	42	51	57	21.9	12.1	<0.0001	0.0005
			esgGal4,UAS-GFP > Jafrac1	205	52.7	48	54	63				
			percent extension		***8.7%***	***14.3%***	***5.9%***	***10.5%***				
		Females	esgGal4,UAS-GFP > +	233	43.5	36	45	54	58.5	61.6	<0.0001	<0.0001
			esgGal4,UAS-GFP > Jafrac1	250	54.8	45	57	66				
			percent extension		***26.0%***	***25.0%***	***26.7%***	***22.2%***				
	B	Males	esgGal4,UAS-GFP > +	117	47.1	42	51	57	10.2	13.7	0.0014	0.0002
			esgGal4,UAS-GFP > Jafrac1	101	54.3	45	57	63				
			percent extension		***15.3%***	***7.1%***	***11.8%***	***10.5%***				
		Females	esgGal4,UAS-GFP > +	119	51.2	39	51	66	16.2	18.5	<0.0001	<0.0001
			esgGal4,UAS-GFP > Jafrac1	123	59.9	54	63	72				
			percent extension		***17.0%***	***38.5%***	***23.5%***	***9.1%***				
	C	Males	esgGal4,UAS-GFP > +	141	47.2	42	48	57	33.4	26.4	<0.0001	<0.0001
			esgGal4,UAS-GFP > Jafrac1	75	55.4	51	63	69				
			percent extension		***17.4%***	***21.4%***	***31.3%***	***21.1%***				
		Females	esgGal4,UAS-GFP > +	139	51.2	39	57	66	22.6	26	<0.0001	<0.0001
			esgGal4,UAS-GFP > Jafrac1	76	63	57	69	75				
			percent extension		***23.0%***	***46.2%***	***21.1%***	***13.6%***				
	**Total**	**Males**	**esgGal4,UAS-GFP > +**	**475**	**47.8**	**42**	**51**	**57**	**61.5**	**47.9**	**<0.0001**	**<0.0001**
			**esgGal4,UAS-GFP > Jafrac1**	**381**	**53.6**	**48**	**57**	**66**				
			percent extension		***12.1%***	***14.3%***	***11.8%***	***15.8%***				
		**Females**	**esgGal4,UAS-GFP > +**	**491**	**47.6**	**36**	**48**	**60**	**70.8**	**85.5**	**<0.0001**	**<0.0001**
			**esgGal4,UAS-GFP > Jafrac1**	**449**	**57**	**48**	**60**	**69**				
			percent extension		***19.7%***	***33.3%***	***25.0%***	***15.0%***				
**esgGFP > Hsp68**	A	Males	esgGal4,UAS-GFP > +	94	50.6	42	54	60	25.4	19.5	<0.0001	<0.0001
			esgGal4,UAS-GFP > Hsp68	102	56.7	51	60	69				
			percent extension		***12.1%***	***21.4%***	***11.1%***	***15.0%***				
		Females	esgGal4,UAS-GFP > +	121	53.1	42	54	66	61	41.5	<0.0001	<0.0001
			esgGal4,UAS-GFP > Hsp68	101	65.4	60	75	81				
			percent extension		***23.2%***	***42.9%***	***38.9%***	***22.7%***				
	B	Males	esgGal4,UAS-GFP > +	86	50	45	54	60	36.4	42.5	<0.0001	<0.0001
			esgGal4,UAS-GFP > Hsp68	91	62.1	57	63	69				
			percent extension		***24.2%***	***26.7%***	***16.7%***	***15.0%***				
		Females	esgGal4,UAS-GFP > +	91	61	54	66	72	77.8	66.2	<0.0001	<0.0001
			esgGal4,UAS-GFP > Hsp68	116	75	69	78	84				
			percent extension		***23.0%***	***27.8%***	***18.2%***	***16.7%***				
	C	Males	esgGal4,UAS-GFP > +	120	50.3	45	51	60	69.7	56	<0.0001	<0.0001
			esgGal4,UAS-GFP > Hsp68	125	60.5	57	66	72				
			percent extension		***20.3%***	***26.7%***	***29.4%***	***20.0%***				
		Females	esgGal4,UAS-GFP > +	122	53	42	54	63	63.4	44.7	<0.0001	<0.0001
			esgGal4,UAS-GFP > Hsp68	104	65.8	57	72	81				
			percent extension		***24.2%***	***35.7%***	***33.3%***	***28.6%***				
	**Total**	**Males**	**esgGal4,UAS-GFP > +**	**300**	**50.3**	**42**	**51**	**60**	**131.7**	**116**	**<0.0001**	**<0.0001**
			**esgGal4,UAS-GFP > Hsp68**	**318**	**59.7**	**54**	**63**	**69**				
			percent extension		***18.7%***	***28.6%***	***23.5%***	***15.0%***				
		**Females**	**esgGal4,UAS-GFP > +**	**334**	**55.2**	**45**	**57**	**66**	**211.4**	**157.1**	**<0.0001**	**<0.0001**
			**esgGal4,UAS-GFP > Hsp68**	**321**	**69**	**63**	**75**	**81**				
			percent extension		***25.0%***	***40.0%***	***31.6%***	***22.7%***				
