# Supplementary material for: Lifespan Extension by Preserving Proliferative Homeostasis in Drosophila
Source: PLoS Genet. 2010 Oct 14;6(10):e1001159. doi: 10.1371/journal.pgen.1001159 (PMC2954830; doi:10.1371/journal.pgen.1001159)
Supplement: Table S6 — Lifespan analysis of flies with moderate expression of Jafrac1 and Hsp68 using the 5961GS driver. Genotypes and lifespan statistics of individual cohorts used for demographic analysis (Figure 6C) are listed. Mean and median lifespan and days at which 25% or 75% of the population were dead are shown for each cohort. Flies from the same population (-RU486 and +RU486) are siblings from individual crosses. Only females are shown. The significance of the changes observed when the flies are raised on RU486 was tested using Log-Rank and Wilcoxon Tests (ChiSquare and p-values). All the analysis was performed using the JMP7 statistical software. (0.34 MB PDF) [file pgen.1001159.s015.pdf]

5961 GeneSwitch > Jafrac1 + Hsp68

Population	Drug	*n*	Mean Lifespan	25% dead	Median Lifespan	75% dead	ChiSquare Log Rank	ChiSquare Wilcoxon	p-value Log Rank	p-value Wilcoxon
A	-RU486	51	66.4	68	75	77	10.8	15.4	0.001	<0.0001
	+RU486	45	74.8	77	80	82				
	percent extension		***12.7%***	***13.2%***	***6.7%***	***6.5%***				
B	-RU486	91	79	79	85	85	52	42	<0.0001	<0.0001
	+RU486	95	87.9	85	87	92				
	percent extension		***11.3%***	***7.6%***	***2.4%***	***8.2%***				
C	-RU486	60	82.6	81	83	85	12.8	12.4	0.0003	0.0004
	+RU486	61	85.3	83	85	90				
	percent extension		***3.3%***	***2.5%***	***2.4%***	***5.9%***				
**Total**	**-RU486**	**203**	**76.9**	**75**	**81**	**85**	**54.8**	**49.4**	**<0.0001**	**<0.0001**
	**+RU486**	**200**	**84.2**	**80**	**85**	**90**				
	percent extension		***9.5%***	***6.7%***	***4.9%***	***5.9%***				
