# Supplementary material for: Lifespan Extension by Preserving Proliferative Homeostasis in Drosophila
Source: PLoS Genet. 2010 Oct 14;6(10):e1001159. doi: 10.1371/journal.pgen.1001159 (PMC2954830; doi:10.1371/journal.pgen.1001159)
Supplement: Text S1 — Description of scoring methods used to monitor intestinal degeneration in aging flies. (0.03 MB DOC) [file pgen.1001159.s016.doc]

**Supplemental Experimental Procedures**

**Description of scoring methods used to monitor intestinal degeneration in aging flies.**

**A.** Scoring of intestinal degeneration using esgGal4,GFP (or esgGal4,GFP; tubGal80^ts^). Representative pictures of the posterior midgut illustrating the four phenotypic classes used to score intestinal degeneration are shown in Figure 1B.

*Individual ISC (blue)*: Guts composed almost entirely of individual stem cells (small GFP-positive cells).

*Intermediate (green)*: Stem cells are primarily found in large clusters (with only a few individual stem cells) and some misdifferentiated cells (large GFP-positive cells) can occasionally be found.

*Strong (yellow)*: Almost all GFP-positive cells are misdifferentiated (very few recognizable stem cells) with major disruption of tissue morphology; however there are still small areas of the gut without the presence of GFP+ cells.

*Severe (red)*: Entire gut is populated by GFP-positive cells (all misdifferentiated cells), and there is a complete loss of normal tissue morphology.

**B.** Scoring of intestinal degeneration using Armadillo (arm) staining (membrane red) and BrdU incorporation (nuclear red). Representative pictures of the posterior midgut from the three phenotypic classes used to score intestinal degeneration are shown in Figure 2B.

*Individual ISC, no BrdU incorporation (blue)*: Guts composed almost entirely of individual stem cells (small, strongly arm-positive cells) with almost no BrdU-positive cell. ECs are arranged in a regular monolayer.

*Moderate dysplasia (white)*: A mixture of individual stem cells (without BrdU incorporation) and misdifferentiated cell clusters containing large, arm-positive cells with some BrdU incorporation.

*Severe dysplasia (yellow)*: intestinal epithelium lmost entirely composed of misdifferentiated cell clusters (very few recognizable individual stem cells) with a majority of BrdU-positive cells.

**C.** Scoring of intestinal degeneration by assessing changes in the anterior midgut epithelium using esgGal4, GFP. Representative pictures of the three categories used to score anterior dysplasia are shown in Figure S6A (white bars mark the limit between the anterior midgut and the proventriculus (PV)).

*Individual ISC (blue)*: no esg^+^ cell clusters or misdifferentiated cells can be observed in the anterior midgut.

*ISC clusters (yellow)*: some esg^+^ cell clusters of more than 3 cells are present in the anterior midgut.

*Proventriculus Invasion (orange)*: esg^+^ cells are observed in the proventriculus.

All scoring was carried out blind.
